# Therapeutic potential of targeting membrane-spanning proteoglycan SDC4 in hepatocellular carcinoma

**DOI:** 10.1038/s41419-021-03780-y

**Published:** 2021-05-14

**Authors:** Heng Yang, Yang Liu, Mei-Mei Zhao, Qiang Guo, Xi-Kang Zheng, Dan Liu, Ke-Wu Zeng, Peng-Fei Tu

**Affiliations:** 1 State Key Laboratory of Natural and Biomimetic Drugs, School of Pharmaceutical Sciences, Peking University, Beijing, 100191 China; 2 Proteomics Laboratory, Medical and Healthy Analytical Center, Peking University Health Science Center, Beijing, 100191 China

**Keywords:** Pharmacodynamics, Receptor pharmacology

## Abstract

Syndecan-4 (SDC4) functions as a major endogenous membrane-associated receptor and widely regulates cytoskeleton, cell adhesion, and cell migration in human tumorigenesis and development, which represents a charming anti-cancer therapeutic target. Here, SDC4 was identified as a direct cellular target of small-molecule bufalin with anti-hepatocellular carcinoma (HCC) activity. Mechanism studies revealed that bufalin directly bond to SDC4 and selectively increased SDC4 interaction with substrate protein DEAD-box helicase 23 (DDX23) to induce HCC genomic instability. Meanwhile, pharmacological promotion of SDC4/DDX23 complex formation also inactivated matrix metalloproteinases (MMPs) and augmented p38/JNK MAPKs phosphorylation, which are highly associated with HCC proliferation and migration. Notably, specific knockdown of SDC4 or DDX23 markedly abolished bufalin-dependent inhibition of HCC proliferation and migration, indicating SDC4/DDX23 signaling axis is highly involved in the HCC process. Our results indicate that membrane-spanning proteoglycan SDC4 is a promising druggable target for HCC, and pharmacological regulation of SDC4/DDX23 signaling axis with small-molecule holds great potential to benefit HCC patients.

## Introduction

Syndecans (SDCs) are a family of transmembrane heparan sulfate proteoglycans ubiquitously expressed on cell surfaces in mammals^1^. SDCs are reported to modulate multiple cell processes, including growth, migration, adhesion, and apoptosis^2–5^. Over the years, it has been found that SDCs are closely related to the occurrence and development of a variety of tumors containing osteosarcoma^6^, breast cancer^7^, and pancreatic cancer^8^. In particular, SDCs are highly involved in HCC development and metastasis. For instance, the decreased expression of SDC1 is a characteristic feature of HCC with high metastatic potential^9^. While cDNA microarray study indicates that the SDC2 gene is overexpressed in HCC tissue^10^. Also, SDC3 is expressed in HCC tissue, especially in tumor stromal vessels, suggesting it may play a fundamental role in HCC tumor angiogenesis^11,12^. Therefore, SDCs have been considered as crucial functional molecules in HCC development and progression.

SDC4 is an important member of SDCs family. SDC4 core protein mainly contains extracellular domain, transmembrane (TM) domain, and cytoplasmic (CP) domain. Extracellular domain allows SDC4 interactions with extracellular matrix proteins through its heparan sulfate (HS) chain and SDC4 core also engage in protein-protein interactions directly^13^. The CP domain, located below the membrane, is thought to interact with cell cytoskeleton, kinases, or other cytokine partners^14^. The function of SDC4 is highly relevant to tumor development and metastasis. Up-regulation of SDC4 has been identified in various malignant tumors such as renal cell carcinoma, melanoma and breast carcinoma^15–17^. Mechanism research showed that SDC4 acted as an FGFR co-receptor to strengthen mitogen-activated protein kinase (MAPK) signaling, which was closely related to cell proliferation and migration^18^. Recently, much attention has been paid to the relationship between SDC4 and HCC. Emerging data demonstrated that SDC4 interacted with actin cytoskeleton to promote the assembly of focal adhesions, finally leading to HCC proliferation and metastasis^19–21^. Moreover, SDC4 has been found to be involved in the regulation of angiogenesis, which is a conspicuous characteristic of HCC development^22,23^. Therefore, SDC4 may be an attractive therapeutic target for HCC. Small molecules selectively targeting SDC4 have never been discovered.

Bufalin is a bioactive compound extracted from the skin and parotid venom glands of toads (*Bufonis Venenum*), which is a traditional Chinese medicine^24^. Clinically, cinobufacini injection, with bufalin as a major active constituent, significantly reduces tumor volume and prolongs survival in patients with advanced HCC. Besides, cinobufacini injection also effectively protects liver function in moderate and advanced primary liver cancer. Experimental studies have shown that bufalin exerts antitumor activities in various cancer cells including HL-60, A549 and HCT116^25–27^. In particular, bufalin induces apoptosis and inhibits migration and invasion in human HCC cell lines^28–31^. Moreover, bufalin exhibits antitumor activities by preventing angiogenesis, reversing drug resistance, and inhibiting tumor invasion and metastasis. Yet, the potential anti-HCC cellular target and mechanism of bufalin remain unclear.

In this study, we for the first time identified SDC4 as a direct anti-HCC cellular target of bufalin in inhibiting cell proliferation, invasion, and angiogenesis. Moreover, we revealed that DEAD-box RNA helicase (DDX23) was a critical SDC4 binding protein for bufalin-regulated anti-HCC effect. Knockdown of SDC4 or DDX23 markedly abolished bufalin-mediated inhibition on HepG2 cell proliferation and migration, potentially by inactivating MMPs and p38/JNK MAPK signaling pathways. Collectively, our study provides new insights into SDC4 as a previously undisclosed promising therapeutic target against HCC, and SDC4/DDX23 signaling axis plays a fundamental role in suppressing HCC growth and progression. Moreover, bufalin may act as the first lead compound directly targeting SDC4 for anti-HCC therapy.

## Materials and methods

### Reagents and antibodies

Bufalin (purity ≥ 98%) was provided by Baoji Herbest Bio-Tech (Baoji, Shanxi, China), solubilized in dimethyl sulfoxide and stored at 4 °C. Biotin-bufalin was synthesized from bufalin (supplementary information). The HCC tissue chip (OD-CT-DgLiv04-001) containing hepatocellular carcinoma tissues and normal tissues was obtained from Shanghai Outdo Biotech Company (Shanghai, China). Rhodamine phalloidin (R415), pierce™ avidin agarose (20219) and supersignal™ west femto maximum sensitivity substrate (34095) were from Thermo Scientific (Pleasanton, CA, USA). 6×Loading buffer (MP006) and RIPA (MP015) were from Macgene (Beijing, China). Heparan sulfate (CAS: 9050-30-0) was from Macklin (Shanghai, China). Antibodies against p-SAPK/JNK (4668), SAPK/JNK (9252), GAPDH (3683), p-P38 MAPK (4511), p-ERK MAPK (4370), MMP2 (4022), MMP9 (3852), P38 MAPK (8690), P53 (9282), p-histone H_2_AX (γH_2_AX, 2577), Ki-67 (9027), N-Cadherin (13116), Vimentin (5741), TCF8/ZEB1 (3396), Snail (3879), E-Cadherin (3195), β-actin (4970) and HRP linked anti-mouse IgG (7076 S) were bought from Cell Signaling Technology (Beverly, MA, USA). Antibodies against Claudin-1 (ab211737) and GPC3 (ab207080) were obtained from Abcam (Cambridge, UK). Antibodies against DDX23 (bs-12199R) was from Bioss (Beijing, China). Antibodies against ERK MAPK (16443-1-AP), SDC4 (11820-1-AP) and HRP-conjugated goat anti-rabbit IgG (SA00001-2) were from Proteintech (Chicago, IL, USA). Antibodies against CDK1 (bs1820), and CyclinB1 (bs1392) were supplied by Bioworld (St. Louis Park, MN, USA).

### Cell culture

Human embryonic kidney 293 cells (HEK293), human hepatoma cells HepG2, Huh7, SK-Hep-1, Hepa3B and human umbilical vein endothelial cells (HUVEC) were provided by Peking Union Medical College, Cell bank, Beijing, China. Cells were maintained in high glucose Dulbecco’s Modified Eagle Medium (DMEM) supplemented with 10% fetal bovine serum (FBS, PAN-Biotech, Aidenbach, Germany) and 1% streptomycin/penicillin. Cells cultured were kept in a humidified incubator with 5% CO_2_ at 37 °C.

### EdU labeling for cell proliferation detection

EdU (5-ethynyl-2′-deoxyuridine) was provided by BeyoClick^TM^ EdU cell proliferation kit with Alexa fluor 488 (Beyotime, Nanjing, Jiangsu, China). EdU stainings were performed following the manufacturer’s instructions. Subsequently, Hoechst 33342 solution was incubated with cells for 10 min at room temperature. Visualized images were captured by fluorescence microscopy (Olympus, Tokyo, Japan). The EdU-positive cell rate was based on the ratio of EdU-positive nuclei (green)/blue fluorescent nuclei.

### Wound healing assay

Cells were allowed to form a confluent monolayer in 6-well plates. A denuded zone with constant width in monolayer cells was created by sterile micropipette tip. Cells were then washed with PBS and exposed to bufalin with the indicated times. The distances of wounds were examined and photographed using a contrast phase microscopy at 0, 24, or 48 h after bufalin treatment. Image J software version 1.48 (NIH, MD, USA) was used to quantify the images of scratch wounds. Cell motility was estimated by calculating percentage of remaining wound area versus initial wound area.

### Cell migration assay

Cell migration was measured in 24-well transwell cell culture chambers (Corning, NY, USA). Cells were seeded into upper chambers at a density of 1×10^5^ cells per well in serum-free DMEM. The lower chambers were filled with DMEM containing 10% FBS as a chemoattractant. After bufalin treatment for 24 or 48 h, the chambers were fixed with 4% formaldehyde for 15 min at room temperature. Non-invading cells were removed from the upper surface using a cotton swab, and chambers from all treatments were stained with 1% crystal violet. Invading cells adhered to underside of chambers were counted under a light microscope, and mean values of five fields were determined.

### Tube formation assay

Tube formation by HUVECs was performed on matrigel (Becton Dickinson, NY, USA). Briefly, matrigel was diluted with an equal volume of FBS-free medium, and seeded into a 96-well plate for polymerization. HUVEC cells were grown onto matrigel and incubated with bufalin for 6 h. Tubular structures were photographed at ×40 magnification. Tube numbers of each well were quantified from five random fields and analyzed by Image J software (ver.1.48).

### Chick chorioallantoic membrane (CAM) assay

Influence of bufalin on angiogenesis was identified by CAM assay. Fertilized chick embryos were obtained from the department of laboratory animal science of Peking university health science center and pre-incubated at 37 °C with 70% humidity. After 3 days, a small hole was perforated on egg shell, and a window was warily created in chick embryo air sac. Clear tape was used to seal the window and the egg was incubated for another 4 days with appropriate humidity and rotation. A piece of sterile filter paper loaded with 150 μL PBS containing bufalin was placed on the top of chorioallantoic membrane. On the 9th day, vessel density and length were observed by injecting appropriate volume of methanol into embryo chorioallantois. Number of angiogenesis was photographed and counted by Image J software (ver.1.48).

### Western blotting analysis

Total proteins were extracted from the cells for indicated treatment by RIPA lysis buffer containing protease inhibitors and centrifuged at 12,000×*g* for 10 min at 4 °C. Protein concentrations were detected by BCA protein assay reagent (TransGen, Beijing, China). Protein samples were transferred to a polyvinylidene difluoride (PVDF) membrane (Millipore, MA, USA) after separating by SDS-PAGE. Membranes were blocked and incubated with primary antibodies overnight at 4 °C. Subsequently, membranes were incubated with HRP-conjugated goat anti-rabbit (1:5000) or anti-mouse IgG (1:1000) secondary antibody. Detection was performed by Tanon-5200 Multi Gel Imaging Analysis System (Tanon, Shanghai, China) and quantified by Gel-Pro Analyzer software version 4.0 (Media Cybernetics, Rockville, MD, USA).

### Immunofluorescence staining

After indicated treatments, cells were fixed with 4% paraformaldehyde, permeabilized in 0.5% Triton X-100, and blocked with 5% BSA. For cytoskeleton detection, 250 μL rhodamine phalloidin working solution (Thermo, Pleasanton, CA, USA) was added to each well. Then, cells were stained with 4,6-diamidino-2-phenylindole (DAPI) solution for 15 min. After incubation with primary antibodies against γH_2_AX (1:400), SDC4 (1:200), DDX23 (1:150), Claudin-1 (1:1000), N-Cadherin (1:200) or avidin-FITC (1:200), cells were exposed to Alexa Fluor 594-labeled secondary antibodies (1:200) and stained with DAPI. Image acquisition was achieved using a confocal laser scanning microscope (Leica, Heidelberg, Baden-Württemberg, Germany).

### siRNA transfection

Cells were grown to 70% confluence before transfection. Afterward, cells were transfected with siRNA using RNAiMAX in Opti-MEM medium (Invitrogen, CA, USA) based on the manufacturer’s protocols. After 6 h, cells were incubated with complete medium for 48 h. The target siRNA sequences against SDC4 and DDX23 (Gene Pharma, Shanghai, China) were presented in Table S1.

### Cellular thermal shift assay (CETSA)

To determine the ligand-target engagement, we performed cellular thermal shift assay (CETSA) according to a previous study^32^. In brief, HepG2 cells were treated with DMSO or 10 nM bufalin for 2 h and collected in PBS supplied with protease inhibitor cocktail. Cells were then heated at indicated temperatures ranging from 40 °C to 64 °C for 3 min on PCR instrument (Bio-Rad, Hercules, CA, USA), followed by repeated freeze-thaw for 5 times in liquid nitrogen. The samples were centrifuged and supernatants were detected by western blotting.

### Drug affinity responsive target stability (DARTS)

Drug affinity responsive target stability assay (DARTS) was adapted from a previous study^33^. HepG2 cells were lysed in 350 μL lysis buffer (0.4% Triton X-100, 400 mM NaCl, 100 mM Tris-HCl, pH 7.5, 20% glycerol) supplemented with protease and phosphatase inhibitors. Lysates were diluted to the same final volume and incubated with bufalin (0, 2.5, 5, 10, and 20 nM) for 1 h at room temperature with shaking gently. Samples were then proteolyzed with pronase (2 μg/mL) for 10 min in reaction buffer (50 mM Tris-HCl, pH 8.0, 50 mM NaCl, 10 mM CaCl_2_). Reactions were terminated by adding SDS-PAGE loading buffer and detected by immunoblot with a specific anti-SDC4 antibody.

### Pull-down assay for bufalin-SDC4 interaction

To confirm the interaction between bufalin and SDC4, pull-down assay was performed according to the previous report^34^. Briefly, biotin-bufalin was first coupled with avidin-agarose beads for 2 h. Cell lysates or heparan sulfates were subsequently incubated with vehicle and bufalin-coupled beads in the absence or presence of bufalin overnight at 4 °C. The beads-captured proteins were separated by SDS-PAGE and detected by western blotting. Heparan sulfate was analyzed by agarose gel electrophoresis.

### Cell cycle analysis

HepG2 cells (2 × 10^5^ cells) were incubated with bufalin (0, 2.5, 5, and 10 nM) for 24 h. Then, cells were harvested, washed and fixed in 70% ethanol at 4 °C. After fixation, cells were washed twice with cold PBS and resuspended in propidium iodide (PI) solution containing RNase. Samples were subsequently analyzed by flow cytometry using a FACS Calibur Flow Cytometer (Becton Dickinson, NY, USA).

### Co-immunoprecipitation (Co-IP) assay

Mammalian expression plasmids encoding HA-tagged SDC4 and mutations (SDC4 ΔC) were constructed with standard molecular biology techniques. For stable isotope labeling with amino acids in cell culture (SILAC), HEK293 cells were cultured in growth medium (SILAC) DMEM (Macgene, Beijing, China), 10% dialyzed fetal bovine serum, penicillin (100 U/mL), streptomycin (100 µg/mL), containing either arginine (Light) and lysine (Light) or heavier isotopic variants of these amino acids (Heavy) and transiently transfected with HA-tagged SDC4 (or SDC4 ΔC) for 48 h. The heavy isotopic-labeled groups were administrated with 10 nM bufalin for 4 h. Both groups were lysed in NP40 lysis buffer (Leagene, Beijing, China) and incubated with anti-HA-tag antibody-conjugated magnetic beads (Cell Signaling Technology, Beverly, MA, USA) for 4 h at 4 °C. The immunoprecipitated proteins were detected by mass spectrometry assay as previously described and subsequently confirmed by immunoblotting^35^.

### Surface plasmon resonance (SPR) analysis

The interaction between bufalin and SDC4 was analyzed with the Biacore T200 system (GE Healthcare, Uppsala, Sweden) at 25 °C. His-tag capture chips (NTA) were used for affinity immobilizations. Activation of the NTA surface was accomplished by injecting a 3 min pulse of NiSO_4_ (200 µM). The recombinant SDC4 at a concentration of 30 μg/mL was applied to achieve non-covalent immobilization at the activated chip surface. The final level of immobilized SDC4 was typically approximately 2700 response units (RU). Various concentrations of bufalin were subsequently injected at a flow rate of 30 μL/min, and PBS-P (10 mM phosphate buffer containing 2.7 mM KCl, 137 mM NaCl, 0.05% surfactant P20 and 5% DMSO) was used as running buffer. The results were analyzed by Biacore evaluation software (T200 version 1.0), and the curve was fitted with a 1:1 binding model.

### Recombinant SDC4 protein expression and purification

The DNA sequence encoding 19-145 amino acids of SDC4 was cloned into pET-28a (+) vector with His-tag between the Nde I and Xho I sites. The recombinant plasmid was transformed into *Escherichia coli* BL21 (DE3) strain and grown at 37 °C in Lysogeny broth (LB) medium supplemented with 100 μg/mL kanamycin. Protein expression was induced by 1 mM isopropyl-β-D-1-thiogalactopyranoside (IPTG) for 16 h at 18 °C. For protein purification, the cells were harvested and suspended in binding buffer (20 mM tris-HCl (pH 7.9), 250 mM NaCl), lysed by sonification, and centrifuged at 14000 × *g* for 30 min at 4 °C. The supernatant was then loaded onto Ni-NTA resin (CWBIO, Beijing, China), washed with 10 mM imidazole, and eluted with 250 mM imidazole. Proteins were concentrated by centrifugal filtration (Amicon Ultra-15, Millipore, USA) with a molecular weight cutoff of 10 kDa. The eluted proteins were analyzed by 10% (w/v) SDS/PAGE and stored in PBS containing 50% (v/v) glycerol at −80 °C.

### MMPs activity detection by gelatin zymography

MMP9 and MMP2 activity in HepG2 cells were detected using matrix metalloproteinase gel electrophoresis kit (Genmed Scientifics, Wilmington, Delaware, USA) according to the manufacturer’s instructions. Briefly, HepG2 cells were washed with PBS and then serum-free culture medium was added before the bufalin (10 nM) exposure. After incubation, the conditioned medium was electrophoresed on 10% SDS-PAGE gels containing 0.1% gelatin. The gels were washed twice with washing buffer (2.5% Triton X-100) for 30 min to remove SDS and were then incubated with reaction buffer (50 mM Tris-HCl, pH 7.6, 200 mM NaCl, 10 mM CaCl_2_) at 37 °C for 42 h. The gels were stained by Coomassie Brilliant Blue R-250 solution (Beyotime, Shanghai, China) and washed with destaining solution (20% methanol and 10% acetic acid in water).

### Protein identification by nano LC-MS/MS

Protein preparation and mass spectrometry analysis**:** HepG2 cells grown in SILAC medium were administrated with 10 nM of bufalin or DMSO for 24 h. Then, cells were harvested and lysed with ice-cold RIPA lysis buffer and quantified using BCA protein assay reagent. The whole-protein lysis was digested with trypsin and filtered by 0.22 μm micro-pore membrane to obtain peptide samples.

Nano-LC-MS/MS analysis was conducted by an LTQ-Orbitrap velos pro-mass spectrometer (Thermo, Pleasanton, CA, USA). EASY LLC II system was adopted to separate the extracted peptides, which were directly autosampled and bound to a trapping column filled with 5 μm RP-C_18_ material. The peptide mixtures were purified on 3 μm RP-C_18_ analytical column (75 μm, 10 cm) and eluted with gradient of H_2_O-ACN (98:2, 60:40, v/v) for 70 min; (60:40, 5:95, v/v) for 5 min; (5:95, v/v) for 20 min. The eluent was injected into mass spectrometer with 300 nL/min. LTQ-Orbitrap mass spectrometer, equipped with a nano-electrospray ion source, was performed in a data-dependent mode. Full scan MS spectra (from *m/z* 350-2000) were obtained from Orbitrap analyzer with a resolution of 60000 (FWHM). In linear ion trap analyzer with a CID of 35% collision energy, top fifteen most abundant precursor ions, which were originated from each MS scan with charge states ≥2, were selected for MS/MS scans. The protein identification was processed and searched by Thermo Proteome Discoverer software version 1.4.

Bioinformatic analysis: analysis of Gene Ontology (GO) protein classification containing biological process (BP), molecular function (MF) and cellular component (CC) was performed with Database for Annotation, Visualization, and Integrated Discovery (DAVID, http://david.abcc.ncifcrf.gov). Kyoto Encyclopedia of Genes and Genomes (KEGG) and ClueGO program, a plug-in Cytoscape software (v.3.5.1) were applied to signaling pathway enrichment analysis^36^.

### Animal studies

Female BALB/c nude mice (Four-week-old) were purchased from the VITAL RIVER Laboratories (Beijing, China) and housed under pathogen-free conditions. HepG2 cells (1 × 10^6^) were injected subcutaneously into the left flank of nude mice. When tumor volumes reached 200 mm^3^, the mice were randomly divided into two groups that each contained five mice. Mice were then treated with bufalin (1 mg/kg) or saline via intraperitoneal (i.p.) injection every day. Tumors were measured with a caliper and volume was calculated using the formula *V* = 1/2 (width^2^ × length). Mice were sacrificed when tumor volumes reached 1000 mm^3^. Tumor tissues were harvested, fixed with fresh 10% neutral formalin, desiccated and embedded in paraffin.

### Immunohistochemistry (IHC)

IHC was performed following standard protocols. Briefly, the HCC tissue chips and sections were dewaxed in xylene and rehydrated to water through descending graded alcohols. Heat-induced antigen retrieval was achieved by incubating in 0.01 M citrate buffer (pH 6) at 90 °C for 20 min. Attenuation of endogenous peroxidases was done by incubation in 3% hydrogen peroxide. Sections were blocked in PBS containing 10% normal goat serum and 0.3% Triton X-100 for 30 min, labeled with Ki-67 (1:400), Claudin-1 (1:250), SDC4 (1:200), DDX23 (1:250) antibodies overnight at 4 °C and incubated with the corresponding goat secondary antibody for 1 h at room temperature. Detection was accomplished using DAB substrate kit (Solarbio, Beijing, China). Slices were then stained with hematoxylin and examined under a light microscope (IX73, Olympus, Japan).

### Statistical analysis

All data were presented as mean±SD (standard deviation) with at least three independent experiments. GraphPad Prism 6.0 software was used for statistical analysis. Student’s *t*-test and one-way analysis of variance (ANOVA) were applied to evaluate the significant difference between different groups. *P* < 0.05 was considered as statistically significant.

## Results

### Bufalin inhibits hepatoma cell proliferation, migration, and angiogenesis

**Fig. 1:**
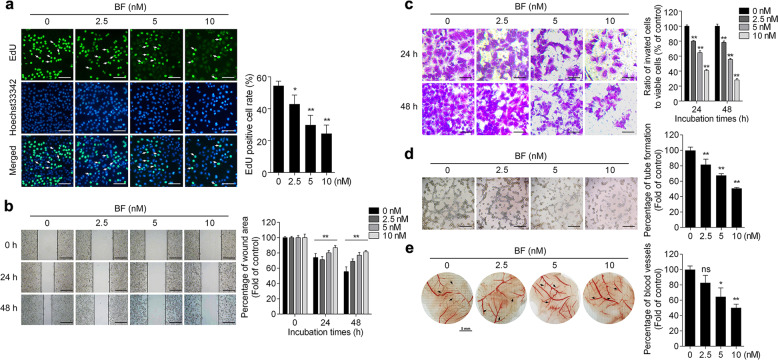
Bufalin inhibits HepG2 proliferation and migration and decreases angiogenesis. **a** Bufalin suppressed the proliferation of HepG2 cells by EdU assay in a concentration-dependent manner. HepG2 cells were treated with bufalin (0, 2.5, 5, and 10 nM) for 24 h. Arrows indicate EdU-positive cells. Number of EdU-positive cells with green fluorescence was counted. **b** Bufalin prevented the migration of HepG2 cells by wound healing assay. **c** Bufalin repressed the invasion of HepG2 cells by transwell chamber assay. Cells stained with crystal violet were counted. **d** Bufalin inhibited tube formation. HUVECs were seeded on matrigel and then administrated with bufalin (0, 2.5, 5, and 10 nM) for 6 h. **e** Bufalin decreased the angiogenesis of CAM. Stroke-physiological saline solution containing bufalin was loaded onto the CAM of developing eggs. Mean areas of blood vessel deviations from 5 embryos in each sample were counted. Data are expressed as mean ± SD for three individual experiments at least. Scale bars = 200 μm (**a**, **b**, **d**), 100 μm (**c**) or 3 mm (**e**). *P < 0.05, **P < 0.01 *vs*. control group; ns, not significant by ANOVA with Student’s *t*-test.

To explore the effect of bufalin on hepatoma cell proliferation, HepG2, Huh7, SK-Hep-1, and Hepa3B cells were treated with various concentrations of bufalin for 24 h and detected by MTT assay. Bufalin-induced proliferative inhibition showed a dose-response in all 4 cell lines studied, with a significant inhibitory effect seen at the lowest dose tested (10 nM) in HepG2 cell (Fig. S1a–d). EdU staining assay was used to investigate cell proliferation of HepG2 and Huh7 cells. Bufalin exhibited a substantially inhibitory effect on HepG2 and Huh7 cells proliferation, indicating an anti-proliferation effect (Fig. 1a and Fig. S1e). Next, we performed scratch wound healing and transwell assays to examine whether bufalin could suppress hepatoma cell migration. As shown in Fig. 1b, the cell-covered areas (gap closure) over time in HepG2 cell were increased by 25.98% ± 5.14% and 44.45% ± 6.23% in un-treatment groups for 24 h and 48 h, respectively. After bufalin (10 nM) treatment, only 13.13% ± 2.25% (24 h) or 18.73% ± 1.78% (48 h) of cell-covered areas were observed. Similar effects of bufalin on Huh7, SK-Hep-1 and Hepa3B cells migration using scratch wound healing assay were also confirmed (Fig. S2a–c). Bufalin also abolished HepG2 and Huh7 cell movements across microporous membranes (Fig. 1c and Fig. S2d), suggesting that bufalin exerts robust suppressive effects on hepatoma cell migration. Furthermore, we conducted tube formation assay to investigate the anti-angiogenic effect of bufalin. Un-treated HUVEC cells formed an organized network on matrigel; however, less intact tube structures and branch points were observed in bufalin-treated cells (Fig. 1d). Moreover, to confirm the anti-angiogenesis effect of bufalin, we performed chick chorioallantoic membrane (CAM) assay to assess the angiogenic activity in vivo. As shown in Fig. 1e, 5 nM and 10 nM of bufalin dramatically decreased blood vessel branch point formation in chicken embryos when compared with control. Overall, these observations suggest that bufalin can effectively suppress hepatoma cell proliferation, migration, and angiogenesis.

### SDC4 is a direct cellular target of bufalin

**Fig. 2:**
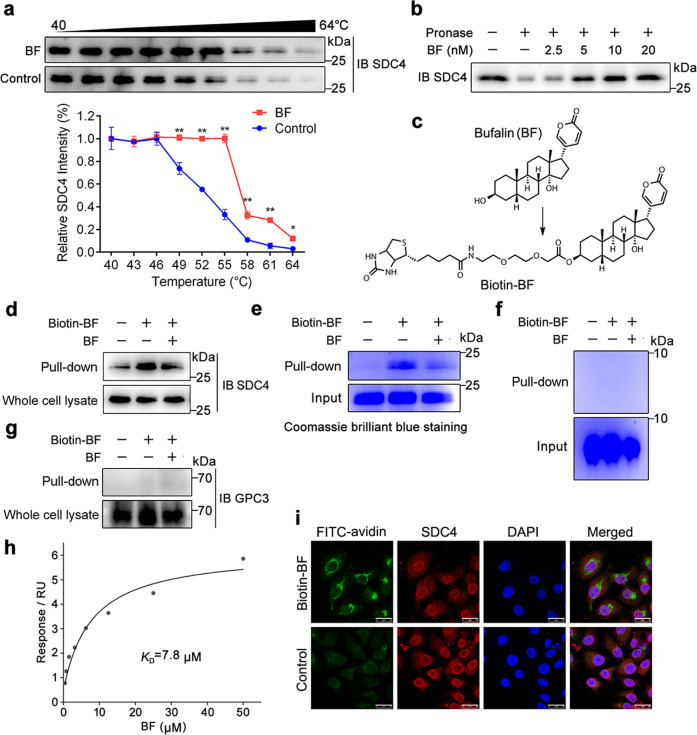
SDC4 is a direct cellular target of bufalin. **a** Bufalin promoted resistance of SDC4 to different temperature gradients, which was detected by CETSA in HepG2 cells. Representative SDC4 western blots and intensity statistics are shown on the panel. **b** Bufalin enhanced SDC4 resistance to proteases, which was investigated by DARTs. **c** Synthesis of biotin-BF. **d** Bufalin specifically interacted with SDC4 in HepG2 cells, which was evaluated by pull-down assay. **e** Bufalin bound to SDC4 recombinant protein by pull-down assay using avidin agarose beads. **f** Pull-down analysis between bufalin and heparin sulfate. **g** Pull-down analysis between bufalin and GPC3. **h** Direct binding between bufalin and SDC4 was verified by SPR analysis. **i** Co-localization of biotin-BF (green) and SDC4 (red) by immunofluorescence (Scale bars = 10 μm). These data are presented as mean ± SD. *P < 0.05, **P < 0.01 vs. control group; ns, not significant by ANOVA with Student’s *t*-test.

Given the inhibitory effects of bufalin on cell migration, invasion, and angiogenesis, we then tried to explore the potential cellular target. Currently, no small molecules have been described to directly target SDC4. According to the literature report, dioscin was a steroidal compound analogous to bufalin in structure, and exerted the inhibitory effect of hepatic stellate cells (HSCs) proliferation and migration through regulating SDC4 expression^37^. Therefore, we speculated that bufalin may likely also regulate SDC4 protein function via direct or indirect interaction. Here, we accidentally observed a ligand-target engagement between bufalin and SDC4. CETSA, a method to assess direct drug binding in cells, showed that bufalin increased SDC4 protein stability in cells when subjected to a temperature gradient (Fig. 2a). In addition, DARTS experiment revealed that bufalin had a potential to stabilize SDC4, resulting in an enhanced susceptibility to proteolysis (Fig. 2b). These observations supported that SDC4 may act as a potential cellular target of bufalin. To further corroborate the direct bufalin binding to SDC4, we synthesized a biotin-labeled bufalin probe (biotin-BF) (Fig. 2c). Pull-down assay coupled with western blot suggested that biotin-BF effectively captured SDC4 from HepG2 and Huh7 cell lysates, which was markedly blocked with an excess amount of bufalin for competition (Fig. 2d and Fig. S3). We also expressed and purified SDC4 recombinant protein (extracellular domain) to confirm the direct interaction of bufalin with SDC4 using pull-down assay (Fig. 2e).

To further exclude the possibility of bufalin combining with heparin sulfate, we performed pull-down experiments using purified heparin sulfate. Results showed that there was no binding between heparin sulfate and bufalin (Fig. 2f). Besides, as a negative control, we also tested the interaction between bufalin and GPC3, which was a cell surface proteoglycan bearing similar heparan sulfate like SDC4. We found no interaction between bufalin and GPC3 (Fig. 2g), indicating that bufalin did not bind to heparan sulfate but specifically targeted SDC4 protein. Moreover, bufalin specifically bound to SDC4 with a dissociation constant (*K*_D_) of 7.8 μM, indicating a strong binding between bufalin and SDC4 (Fig. 2h). Next, we performed double immunofluorescence assay to explore the co-location of SDC4 and biotin-bufalin (BF) in HepG2 cells. As shown in Fig. 2i, SDC4 (red) showed obvious fluorescence overlap (yellow) with biotin-BF (green) to elegantly demonstrate a direct bufalin-SDC4 interaction in cells. Collectively, these results indicate that SDC4 is a direct cellular target of bufalin.

### Bufalin promotes SDC4 interaction with DDX23 to regulate genomic instability

**Fig. 3:**
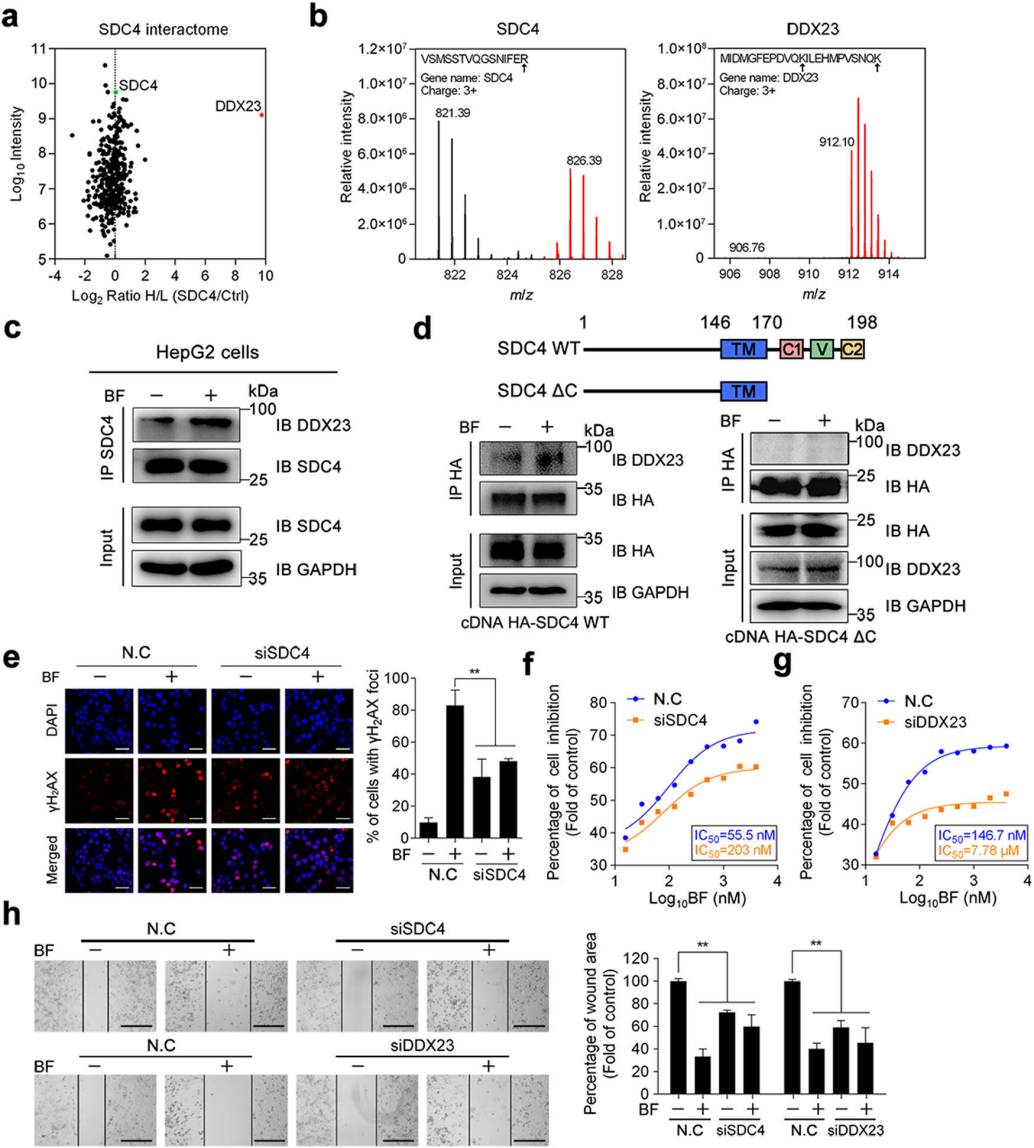
Bufalin promotes SDC4 interaction with DDX23 to regulate genomic instability. **a** Scatter plot depicted SDC4 interactome. Log_2_ heavy (H) / light (L) ratios of the quantified proteins were shown on *x*-axis and log_10_ signal intensity (combined for heavy and light peptides) on the *y*-axis. **b** Quantification of SDC4 and DDX23 by 2-D HPLC LTQ/Orbitrap MS. Peaks with light-labeled (gray) and heavy-labeled (red) peptides were presented. VSMSSTVQGSNIFER and MIDMGFEPDVQKILEHMPVSNQK were peptides from SDC4 and DDX23, respectively. (R, Arginine; K, Lysine) *m*/*z*, mass/charge ratio. **c** Bufalin promoted the SDC4 binding to DDX23. Western blot of co-immunoprecipitation was performed with anti-SDC4 or anti-DDX23 antibodies. **d** Structure outline of SDC4 (upper panel). SDC4 interacts with DDX23 through its 1-170 amino acids peptide (lower panel). **e** Bufalin increased the number of γH2AX foci, which was blocked by siSDC4. Negative control or siSDC4 HepG2 cells were treated with/without bufalin, followed by γH2AX foci and DAPI staining. **f**, **g** siSDC4 or siDDX23 reversed bufalin-dependent cell viability decrease. Cells transfected with siSDC4, siDDX23 or negative control were treated with bufalin for 24 h. Cell viability was measured by MTT assay. **h** Cells transfected with siSDC4, siDDX23 or negative control were incubated with/without bufalin (10 nM) for 48 h. Wound area was calculated. Data are representative of three independent experiments. *P < 0.05, **P < 0.01 *vs*. control group; ns, not significant by ANOVA with Student’s *t*-test.

To further clarify potential pharmacological mechanism of bufalin on cell migration and invasion, we attempted to identify the interaction partners of SDC4 in cells. We conducted co-IP assay in HA-tagged SDC4 overexpression HEK293 cells using SILAC-based quantitative proteomics. SDC4, together with interaction partner proteins, were immunoprecipitated with anti-HA-tag antibody and identified by mass spectrometry. A total of 149 SDC4-binding proteins were identified, and 14 proteins displayed significantly enhanced interactions with SDC4 upon bufalin treatment (Fig. 3a, b). Next, we conducted co-IP experiments to confirm whether bufalin could promote the complex formation of endogenous SDC4 with DDX23 in HepG2 cells. Our results showed that endogenous SDC4 successfully precipitated DDX23 in HepG2 cells, and bufalin treatment enhanced the interaction between SDC4 and DDX23 (Fig. 3c). Meanwhile, immunofluorescence staining showed that bufalin treatment substantially induced the superposition of red fluorescence (DDX23) and green fluorescence (SDC4) in HepG2 cell (Fig. S4), implying that bufalin could promote the interaction of SDC4 with DDX23. Since SDC4 mainly contains a transmembrane (TM) domain and a cytoplasmic (CP) domain, and SDC4 normally interacts with its interacting partners through its CP domain, we then explored whether SDC4 interacts with DDX23 through its CP domain. As shown in Fig. 3d, we found that CP domain is essential for SDC4-DDX23 interaction. Particularly, the deletion of CP domain on SDC4 (ΔC) completely abolished the SDC4-DDX23 interaction, indicating that SDC4 forms a complex with DDX23 through its CP domain.

Remarkably, DDX23 has been discovered as a component of the U4/U6-U5 tri-snRNP complex involved in pre-mRNA splicing in cancer progression^38^. Dysfunction or absence of DDX23 leads to massive genomic instability, which is characterized by high levels of DNA double-strand breaks (DSBs)^39^. To gain further insights into the function relationship between SDC4 and DDX23, we depleted SDC4 from HepG2 cells and measured the amount of damaged DNA. As expected, bufalin significantly increased nuclear γH2AX foci, a marker for DNA double-strand breaks, which was noticeably blunted following SDC4 depletion (Fig. 3e). Besides, bufalin-mediated inhibition on cell proliferation and migration was substantially impaired after SDC4 or DDX23 depletion (Fig. 3f–h). Collectively, these results strongly suggest that bufalin promotes SDC4 interaction with DDX23 to regulate genomic instability through inducing DSBs.

### Quantitative proteomics-coupled with bioinformatics reveals the landscape of bufalin-regulated signaling networks

Next, we tried to identify the fundamental signaling pathways that were mediated by bufalin using quantitative proteomic profiling in isotope-labeled HepG2 cells. Nano-LC-MS/MS system for quantitative proteomics was adopted to identify differentially altered proteins landscape in cells. The data from Thermo Proteome Discoverer database showed that a total of 5682 proteins were reproducibly detected among three biological replicated experiments. For further statistical and bioinformatics analyses, we performed student’s *t*-tests to identify differentially expressed proteins that were statistically significant. Notably, the proteins with a more than 1.25 fold increase or decrease were filtrated. Specifically, 172 differentially expressed proteins were identified, and 64 (1.1% of the proteome) of which were markedly up-regulated and 108 (1.9% of the proteome) were markedly down-regulated upon bufalin treatment.

**Fig. 4:**
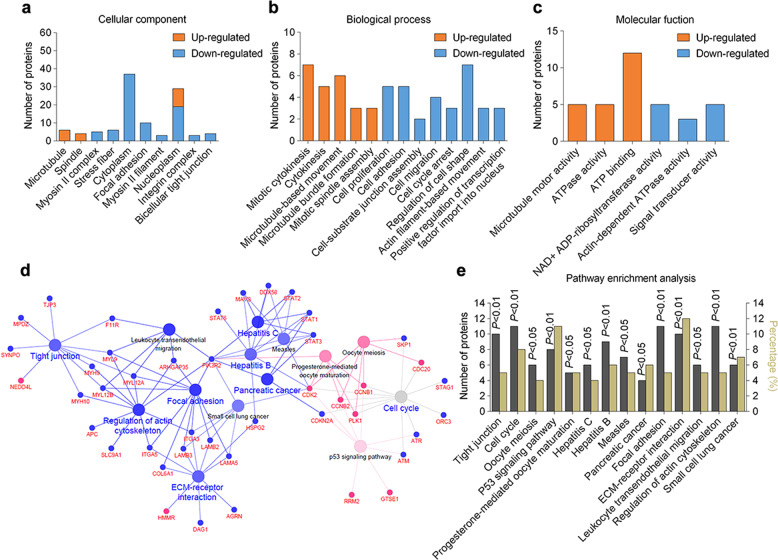
Bioinformatics analysis reveals the landscape of bufalin-regulated signaling networks. **a** Distribution of cellular component categories for differential proteins significantly altered by bufalin. **b** Distribution of biological process categories for differential proteins substantially affected by bufalin. **c** Distribution of molecular function categories for differential proteins dramatically changed by bufalin. **d** Network diagram of pathway enrichment analysis for bufalin-altered proteins in HepG2 cells. Blue dots represented markedly down-regulated proteins/pathways and red dots indicated significantly up-regulated proteins/pathways. **e** Statistical analysis of pathway enrichment. Gray columns plotting on the left *Y*-axis depicted the number of identified proteins found in each pathway. Yellow columns plotting on the right *Y*-axis depicted the percentage of identified proteins over total proteins.

To characterize the impact of bufalin on proteomic changes, these differentially expressed proteins were annotated and categorized according to GO terms enrichment analysis. As illustrated in Fig. 4a, the major cellular localizations of these proteins were cytoplasm, nucleoplasm, and focal adhesion. Moreover, these proteins were involved in multiplied biological processes including cell proliferation, cell migration, cell shape, mitotic cytokinesis, and microtubule-based movement (Fig. 4b). Functionally, these proteins possessed diversified activities, such as ATP binding, microtubule motor activity and signal transducer activity (Fig. 4c). Therefore, our findings indicate that bufalin is widely involved in multiple biological processes associated with cell proliferation, migration, and cytoskeleton regulation.

Further analysis of pathways and pharmacological networks influenced by bufalin was performed by KEGG pathway enrichment with Cytoscape. A variety of pathways including actin cytoskeleton, cell cycle, P53 signaling pathway, tight junction, focal adhesion, and ECM-receptor interaction were markedly altered by bufalin treatment (Fig. 4d). Notably, the percentage of identified proteins over the total proteins in P53 signaling pathway and ECM-receptor interaction pathway tend to dominate in significantly enriched pathways (Fig. 4e), indicating that SDC4 may contribute to bufalin-mediated signaling pathway in focal adhesion and ECM-receptor interaction in cytoskeleton organization^40–42^. Together, these findings suggest that bufalin effectively regulates multiple cell proliferation and migration-associated pathways in the cell cycle, actin cytoskeleton, and ECM-receptor interaction.

### Bufalin blocks MMPs, MAPK, and EMT signaling pathways in HepG2 cells

**Fig. 5:**
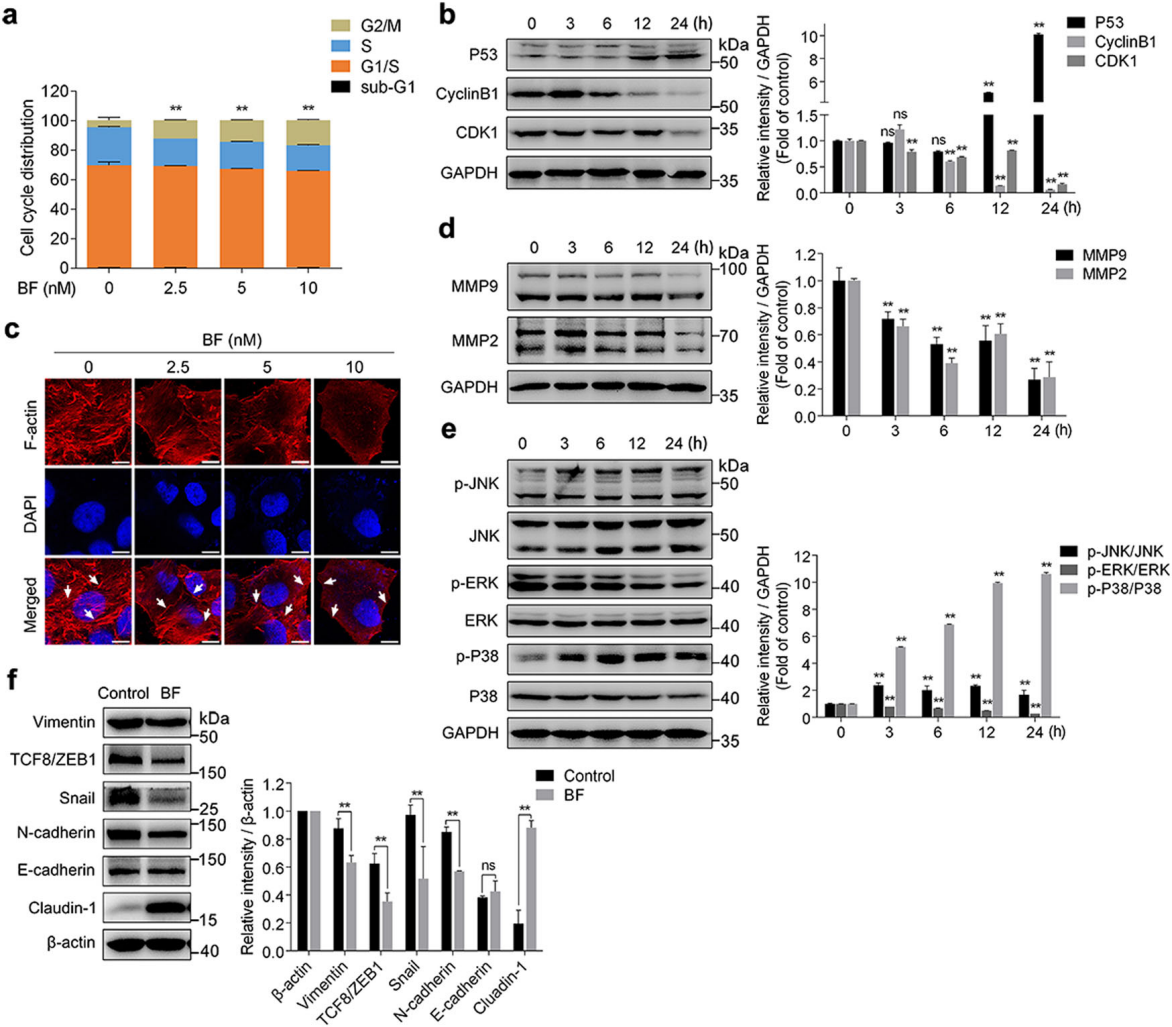
Bufalin blocks MMPs, MAPK and EMT signaling pathways in HepG2 cells. **a** Bufalin-induced G2/M phase arrest in HepG2 cells. The cell cycle distribution was determined by flow cytometric analysis and cell cycle distribution was quantified. **b** Bufalin decreased the protein expression of CDK1, cyclinB1 and increased the protein expression of P53. Cells were exposed to bufalin (10 nM) for indicated time points and protein levels were detected by western blot. **c** Bufalin inhibited the formation of F-actin cytoskeleton in HepG2 cells. Arrows indicated F-actin (red) stained with rhodamine-phalloidin (Scale bars =10 μm). **d** Bufalin decreased the protein expression of MMP2 and MMP9. Cells were treated with or without bufalin (10 nM) for indicated time points. **e** Bufalin inhibited ERK/JNK/P38 MAPK signaling cascades. Non-phosphorylations/phosphorylations of ERK, JNK, and p38 were detected by western blot. **f** Bufalin inhibited EMT signaling pathway. Cells were treated with or without bufalin (10 nM) for 24 h. The levels of Vimentin, TCF/ZEB1, Snail, N-cadherin, E-cadherin, Claudin-1 were evaluated by western blot. Data are representative of three independent experiments. **P < 0.01 vs. control group; ns, not significant by ANOVA with Student’s *t*-test.

Bioinformatics analysis suggested that bufalin had a significant regulatory effect on the cell cycle. We thus investigated the distribution of the cell cycle in HepG2 cells upon bufalin treatment. Result showed bufalin induced a pronounced G2/M phase arrest in HepG2 cells (Fig. 5a and Fig. S5a). Moreover, we inspected G2/M phase-specific protein expression. As illustrated in Fig. 5b, bufalin caused an increase of P53 and down-regulated Cyclin A, Cyclin B1, and CDK1 expression in a time-dependent manner. We next examined the changes of F-actin organization by fluorescent rhodamine-phalloidin staining assay. As shown in Fig. 5c, untreated cells showed regular and well-defined actin organization, while bufalin-treated cells displayed a significantly decreased and diffused F-actin disposition. Also, the expression of MMP2 and MMP9 was dramatically down-regulated in bufalin-treated cells (Fig. 5d). Further, the gelatin zymography assay showed that bufalin exerted a significant inhibitory effect on MMP2 activity, indicating that suppression of MMP2/9 expression and MMP2 activity may contribute to bufalin-mediated inhibition on cell migration (Fig. S5b).

Bioinformatics analysis also provided a hint for MAPK signal transduction pathway. In our study, we observed that bufalin significantly increased the phosphorylation levels of JNK and P38, and decreased ERK phosphorylation in a time-dependent manner (Fig. 5e). Additionally, bufalin elevated Claudin-1 and decreased Vimentin, TCF8/ZEB1, Snail and N-cadherin expression, which were involved in epithelial-mesenchymal transition (EMT) signaling pathway (Fig. 5f). Also, we performed immunofluorescence assay for detecting the expression of Claudin-1 and N-cadherin, which were specific markers of EMT. Our results showed that bufalin significantly increased Claudin-1 expression and decreased N-cadherin protein level (Fig. S5c, d). Taken together, these results indicate that bufalin exerts anti-HCC effect through blocking cell cycle and disrupting MMPs, MAPK and EMT signaling pathways.

### SDC4/DDX23 axis is crucial for driving HepG2 proliferation and migration signaling pathways

Given the crucial role of SDC4/DDX23 complex on cell proliferation and migration, we evaluated the expression of SDC4 and DDX23 proteins in HCC tissue from clinical patients. Immunohistochemistry staining results showed that both SDC4 and DDX23 had high expression levels in HCC tissues compared with adjacent normal tissues (Fig. S6a, b). There was a significant correlation between SDC4 expression and clinicopathological stages in all HCC tissue specimens (*P* < 0.05), with no significant correlations in age, gender and hepatitis (all *P* > 0.05). Moreover, DDX23 expression share the same correlation with clinicopathological features of HCC (Table S2). According to Pearson’s correlation test, 20 cases of positive expression and 13 cases of negative expression of SDC4 and DDX23 were obtained in a total of 41 cases of HCC tissues, suggesting a positive correlation between SDC4 and DDX23 (*r* = 0.598, *P* < 0.001) (Table S3). These data demonstrate that SDC4 and DDX23 played an essential and unique role in HCC tumor proliferation and development.

**Fig. 6:**
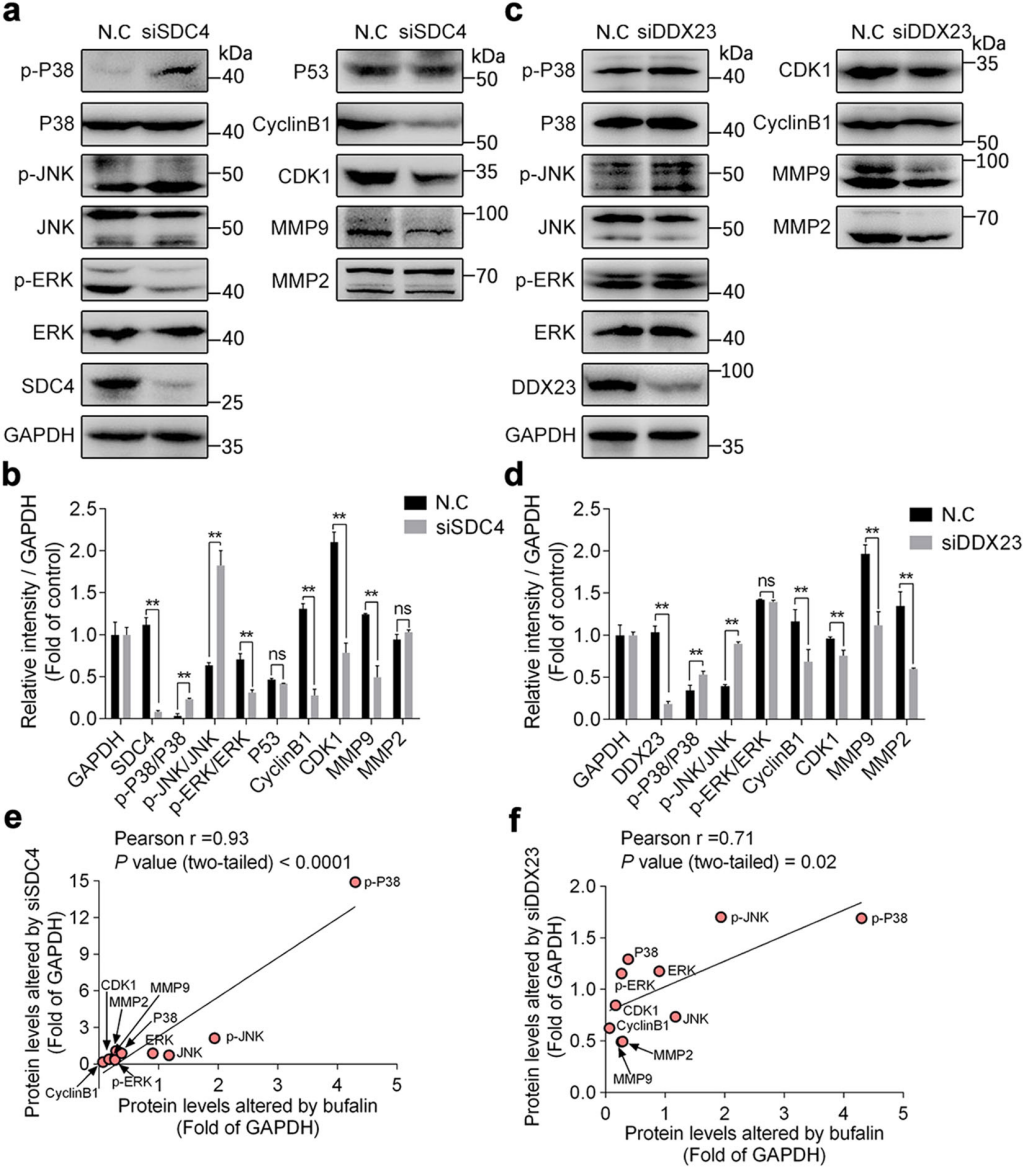
SDC4/DDX23 axis is crucial for driving HepG2 proliferation and migration. **a**, **b** siSDC4 blocked ERK/JNK/P38 MAPK, cell cycle, and MMP9 signaling pathways. CDK1, CyclinB1, P53, MMP2/9 and non-phosphorylations/phosphorylations of ERK/JNK/P38 were determined by western blot in SDC4 knockdown HepG2 cells. **c**, **d** siDDX23 prevented JNK/P38 MAPK, cell cycle, and MMP2/9 signaling pathways. CDK1, CyclinB1, MMP2/9 and non-phosphorylations/phosphorylations of ERK/JNK/P38 were detected by western blot in DDX23 knockdown HepG2 cells. **e** Correlation analysis between bufalin-regulated and siSDC4-altered proteins in HepG2 cells. **f** Correlation analysis between bufalin-regulated and siDDX23-altered proteins in HepG2 cells. Each data point represented the protein level (CDK1, CyclinB1, MMP2/9, and non-phosphorylations/phosphorylations of ERK/JNK/P38) changed by both bufalin and siSDC4 or siDDX23 treatment. Statistical significance was measured by Pearson’s correlation test. Data are expressed as mean ± SD for three individual experiments. **P < 0.01 vs. control group; ns, not significant by ANOVA with Student’s *t*-test.

Then, we put forward a hypothesis that SDC4/DDX23 might act as an upstream regulator for cell cycle, MAPK and MMPs signaling pathways. First, we knocked down SDC4 and DDX23 in HepG2 cells by transfecting siRNAs and significantly decreased SDC4 and DDX23 protein expression (Fig. S7a–d). Next, we examined MMPs, MAPK and EMT signaling pathways in SDC4 or DDX23 knockdown HepG2 cells. As shown in Fig. 6a–d and Fig. S8, phosphorylations of P38, JNK and Claudin-1 were markedly augmented, and meanwhile, CDK1, CyclinB1, and MMP9 protein expression was reduced both in SDC4 and DDX23 knockdown HepG2 cells. This result underlined the importance of SDC4 and DDX23 on MAPK activation, cell cycle, EMT, and migration-associated proteins. Moreover, we noted that SDC4 knockdown significantly decreased phosphorylation level of ERK, which was not affected in DDX23 knockdown HepG2 cells (Fig. 6a–d), and may be attributed to the possibility that ERK is phosphorylated by FAK in SDC4/PKCα/FAK/ERK1/2 bypass pathway^43^. Further, Pearson’s correlation analysis of linear relationship between bufalin-regulated and siDDX23 (siSDC4)-altered proteins was performed. Result showed that the protein expressions regulated by siSDC4 or siDDX23 were both positive correlated with bufalin treatment (Fig. 6e, f), suggesting that SDC4 and DDX23 are vital molecules for bufalin-mediated antitumor signals. Finally, SDC4 or DDX23 knockdown cells were treated with bufalin and the expression of P53, MMP2/9, Cyclin B1, CDK1, JNK was detected by western blotting. Results showed that bufalin could not induce obvious changes on these proteins expression upon siSDC4 or siDDX23 transfection (Fig. S9), indicating that SDC4 and DDX23 play crucial roles for bufalin-dependent anti-HCC effect. Collectively, our data indicate that SDC4/DDX23 axis is responsible for driving HepG2 proliferation and migration through fine-tuned regulation of cell cycle, MMPs, EMT, and MAPK signaling pathways.

### Bufalin inhibits HCC proliferation in vivo

**Fig. 7:**
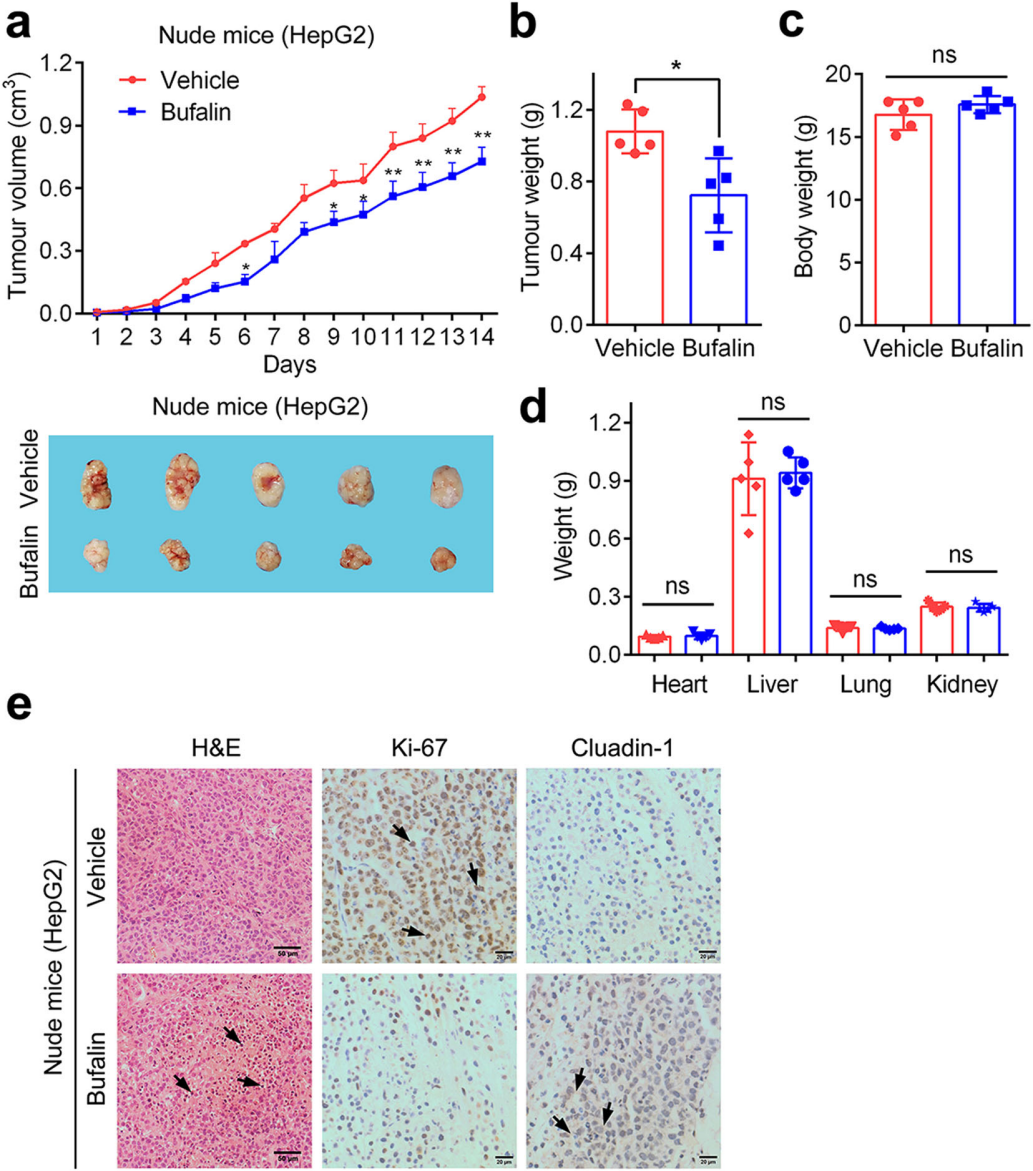
Bufalin inhibits HCC proliferation in vivo. **a** Bufalin decreased tumor volume in nude mice transplanted with HepG2 cells (mean ± SD, *n* = 5). **b** Bufalin reduced tumor weight in HepG2 xenograft mice compared with vehicle treatment. Red dots represent vehicle and blue dots denote bufalin treatment group. **c** Bufalin treatment did not significantly alter the body weight of HepG2 xenograft mice. Red dots symbolize vehicle and blue dots represent bufalin treatment group. **d** Bufalin treatment had no significant effect on the weight of heart, liver, lung, and kidney in HepG2 xenograft mice. Red dots represent vehicle and blue dots stand for bufalin treatment group. **e** H&E staining (left) was performed in tumors that developed in the vehicle and bufalin treatment groups (Scale bars = 50 μm). Black arrows denote apoptotic cells. Immunohistochemistry (IHC) analysis of Ki-67 and Claudin-1 (right) expression in the vehicle and bufalin treatment groups (Scale bars = 20 μm). Black arrows indicate Ki-67 and Claudin-1 positive staining cells. *P < 0.05, **P < 0.01 vs. control group; ns, not significant by ANOVA with Student’s *t*-test.

Based on in vitro experiments, we next explored the inhibitory effect of bufalin on the growth and proliferation in HepG2 cell tumor xenograft. From Fig. 7a, b, bufalin substantially reduced the tumor volume and weight in nude mice transplanted with HepG2 cell compared with control group. The body and organ weights were not significantly altered, indicating that bufalin has no obvious side effects (Fig. 7c, d). In addition, H&E staining showed that tumor cells in control group featured clear and regular nuclei with prominent nucleoli. The cytoplasm was characteristically pink and clear. However, after bufalin treatment, the tumor nuclei shrank dramatically and condensed with decreased nuclear/cytoplasm ratio (Fig. 7e), indicating that bufalin promoted tumor cell apoptosis in HCC xenograft model. Further, the protein levels of EMT markers Claudin-1 and proliferation marker Ki-67 were determined by IHC staining. Results showed that bufalin treatment markedly decreased the protein levels of Ki-67, and increased the expression of Claudin-1 in tumor tissues (Fig. 7e), suggesting that bufalin inhibited tumor proliferation and impede EMT signaling pathway in HCC xenografts. Taken together, all these data demonstrate that bufalin can inhibit HCC proliferation in vivo with no significant side effects.

## Discussion

SDCs have been reported as prognostic indicators and therapeutic targets in malignancies. Single mAbs or drug-conjugated antibodies targeting SDC1 have been successfully used in melanoma, triple negative breast cancer and multiple myeloma treatment in vitro and in vivo studies^44^. However, there are few reports about small-molecule drugs targeting SDCs for cancer therapy. The development of novel SDCs inhibitors will extend the biological roles of SDCs in pharmacological targeting that will further improve the efficacy of established therapeutic approaches. In this study, we investigated the underlying repression mechanism of bufalin on HCC migration and invasion. Encouragingly, we discovered that bufalin specially bond to SDC4 as a direct cellular target to prevent HepG2 cell proliferation, invasion and decrease angiogenesis. Further, we revealed DDX23 as an essential regulator of SDC4 in driving cell proliferation and migration signaling pathways. These findings indicate that SDC4 is a promising druggable target for HCC treatment, and bufalin may represent a first-in-class anti-HCC small-molecule through targeting SDC4.

To invade adjacent tissues, cancer cells have to escape from tumor mass by breaking intercellular adherent junctions, and focal adhesion is dynamic during wound healing and cell migration. SDC4 has been described as a contributing factor to focal adhesion and possesses transmembrane signal regulatory activity on FGF2, *β*1 integrin, and cytoskeletal modifications^45,46^. These signals are crucial determinants of tumorigenesis and angiogenesis. However, small molecules targeting SDC4 have never been reported. Our present study described the first small-molecule bufalin that directly binds to SDC4 for effective inhibition on MMPs, p38/JNK MAPK and EMT signal cascades through forming SDC4/DDX23 complex. Thus, bufalin served as a novel molecular template for anti-HCC agent development by targeting SDC4.

It is important to note that DDX23 dysfunction causes excess genomic perturbations and results in numerous DSBs to disrupt cell proliferation and migration. A similar phenomenon was observed both in bufalin-treated and DDX23 knockdown HepG2 cells. We speculated that bufalin directly induced SDC4 interaction with DDX23 by promoting its cytoplasm translocation from the nucleus. Interestingly, the chemical-induced DDX23 dysfunction by bufalin was phenocopied in siDDX23 or siSDC4-induced HepG2 cells, followed by cell proliferation and migration inhibition, indicating solid evidence for a linkage between bufalin and SDC4/DDX23 signaling axis.

SDC4 has been found to regulate a plethora of function proteins such as Fn, antithrombin-1 as well as heparin-binding growth factors including fibroblast growth factors (FGFs), midkine and pleitrophin, and laminin^47–49^. However, we still poorly understood the signaling pathway associated with bufalin-mediated SDC signal cascade. Here, we found that DDX23, a component of U4/U6-U5 tri-snRNP complex involved in pre-mRNA splicing, strongly interacted with SDC4 upon bufalin treatment. It has been previously reported that DDX23 dysfunction can result in R-loops accumulation and genomic instability which is characterized by DSBs^39^. DSBs are the most serious chromosome damage at the genomic level in mammalian cells^50,51^. Herein, we found that bufalin induced a great amount of DSBs, which was reversed by SDC4 knockdown in HepG2 cells. We speculate that these DSBs may accumulate in the promoters of a set of cell cycle genes including cyclinB1 and CDK1, and MMP9. Moreover, we observed that the EMT marker Claudin-1 and P53 expression was significantly up-regulated by bufalin. A putative mechanism may be that bufalin-dependent DSBs suppress the expression of some unique function proteins including ubiquitin ligases or lysosomal proteins for specific Claudin-1 and P53 degradations, which still remains to be elucidated.

In summary, we identified SDC4 as a direct cellular target of small-molecule bufalin for HCC treatment. Moreover, the SDC4/DDX23 axis is crucial for driving HepG2 proliferation and migration through regulating cell cycle, MMPs, and MAPK signaling pathways. These findings suggest that SDC4 is a promising anti-HCC target and provides important clues for new drugs discovery and development.

## Supplementary information

Supplementary Material

Supplementary Material

Supplementary Material

Supplementary Material

Supplementary Material

Supplementary Material

Supplementary Material

Supplementary Material

Supplementary Material

Supplementary Material

Supplementary Material

Supplementary Material

Supplementary Material

Supplementary Material

Supplementary Material

Supplementary Material

Supplementary Material

Supplementary Material

Supplementary Material

Supplementary Material
