# Supplementary material for: Therapeutic potential of targeting membrane-spanning proteoglycan SDC4 in hepatocellular carcinoma
Source: Cell Death Dis. 2021 May 14;12(5):492. doi: 10.1038/s41419-021-03780-y (PMC8121893; doi:10.1038/s41419-021-03780-y)
Supplement: Supplementary file 1 — Supplementary Material [file 41419_2021_3780_MOESM1_ESM.docx]

**Therapeutic potential of targeting membrane-spanning proteoglycan SDC4 in hepatocellular carcinoma**

**I. Supplementary methods**

**Synthesis of biotin-bufalin**

Compound **C3**: Bufalin (200 mg, 0.517 mmol, 1 eq.), DCC (128 mg, 0.621 mmol, 1.2 eq.), and DMAP (6.3 mg, 0.052 mmol, 0.1 eq.) were dissolved with 10 mL DCM. To this solution, compound **C2** (300 mg, 0.778 mmol, 1.5 eq.) was added. The reaction mixture was stirred at room temperature overnight and filtrated to remove insoluble impurities. The filtrate was evaporated under reduced pressure to gain crude product, which was subsequently purified by flash column chromatography on silica gel and acquired compound **C3** (456 mg, 91.2% yield) as a white powder. ^1^H NMR (500 MHz, CDCl_3_) *δ* 7.82 (dd, *J* = 8.5, 2.0 Hz, 1H), 7.75 (d, *J* = 7.5 Hz, 2H), 7.60 (d, *J* = 7.0 Hz, 2H), 7.38 (t, *J* = 7.5 Hz, 2H), 7.30 (t, *J* = 7.5 Hz, 2H), 7.21 (d, *J* =1.5 Hz 1H), 6.24 (d, *J* = 9.5 Hz, 1H), 5.52 (brs, 1H), 5.18 (s, 1H), 4.36 (d, *J* = 7.0 Hz, 2H), 4.21 (t, *J* = 7.0 Hz, 1H), 4.12 (s, 2H), 3.73 (s, 2H), 3.67 (s, 2H), 3.59 (t, *J* = 5.0 Hz, 2H), 3.45 (s, 1H), 3.43 – 3.38 (m, 2H), 2.43 (dd, *J* = 9.5, 7.0 Hz, 1H), 2.19 – 2.13 (m, 1H), 2.04 – 1.99 (m, 1H), 1.94 (s, 1H), 1.89 – 1.82 (m, 2H), 1.75 – 1.67 (m, 3H), 1.67 – 1.60 (m, 3H), 1.53 (s, 1H), 1.46 – 1.41 (m, 2H), 1.40 (s, 1H), 1.35 (s, 1H), 1.33 (s, 1H), 1.30 (d, *J* = 3.4 Hz, 1H), 1.24 (s, 1H), 1.22 (s, 1H), 0.92 (s, 3H), 0.67 (s, 3H).

Compound **C4**: To a stirred mixture of **C3** (456 mg, 0.6 mmol, 1 eq.) and DMF (5 mL), piperidine (100 μL) was added in one portion. The reaction was stirred at room temperature for 4 h. The mixture was concentrated under reduced pressure to gain crude product **C4** (67.4 mg, 14.78% yield), which was used in the next step without further purification. ^1^H NMR (500 MHz, CDCl_3_) *δ* 7.88 (dd, *J* = 9.5, 1.5 Hz, 1H), 6.28 (d, *J* = 10.0 Hz, 1H), 5.22 (s, 1H), 4.17 (s, 2H), 3.78 – 3.70 (m, 4H), 3.55 (t, *J* = 4.5 Hz, 2H), 2.90 (s, 2H), 2.52 – 2.46 (m, 1H), 2.25 – 2.18 (m, 1H), 2.11 – 2.05 (m, 1H), 1.97 – 1.86 (m, 3H), 1.78 – 1.64 (m, 8H), 1.56 (m, 4H), 1.40 – 1.26 (m, 5H), 0.98 (s, 3H), 0.73 (s, 3H) . HRMS *m*/*z* 532.3272 [M + H]^+^.

Compound **C6**: This compound was synthesized according to procedure described by Andrea B with a slight modification^1^. Biotin (**C5**, 1 g, 4.1 mmol, 1 eq.), *N*-hydroxysuccinimide (0.944 g, 8.2 mmol, 2 eq.), and EDC⋅HCl (1.572 g, 8.2 mmol, 2 eq.) were dissolved in 50 mL of anhydrous DMF. The reaction mixture was stirred at 50 °C for 8 h. The solvent was removed under reduced pressure and washed with MeOH to give 1.56 g of **C6** (80% yield) as a white powder. ^1^H NMR (500 MHz, CD_3_SOCD_3_) *δ* 6.41 (s, 1H), *δ* 6.35 (s, 1H), 4.34 – 4.27 (m, 1H), 4.18 – 4.11 (m, 1H), 3.12 – 3.10 (m, 1H), 2.81 (s, 3H), 2.67 (t, *J* = 7.5 Hz, 2H), 2.58 (d, *J* = 12.5 Hz, 1H), 1.74 – 1.57 (m, 3H), 1.55 – 1.38 (m, 3H).

Compound **C7**: Compounds **C4** (67.4 mg, 0.127 mmol, 1 eq.) and **C6** (64.8 mg, 0.189 mmol, 1.5 eq.) were dissolved in 5 mL DMF. To this solution, 65.5 μL DIPEA (0.379 mmol, 3 eq.) was added. The reaction mixture was stirred at room temperature overnight. The solvent was removed in a vacuum. The crude product was purified by flash column chromatography on silica gel and yielded compound **C7** (47.66 mg, 36%). ^1^H NMR (500 MHz, CD_3_OD) *δ* 8.03 (d, *J* = 9.5 Hz, 2H), 5.22 (s, 1H), 4.55 – 4.52 (m, 1H), 4.37 – 4.33 (m, 1H), 4.21 (s, 2H), 4.13 (m, 1H), 3.75 (s, 2H), 3.68 (s, 2H), 3.59 (t, *J* = 5.0 Hz, 2H), 3.43 – 3.36 (m, 3H), 2.97 (dd, *J* = 12.5, 4.5 Hz, 1H), 2.61 – 2.57 (m, 1H), 2.27 (m, 3H), 2.07 (d, *J* = 18.2 Hz, 2H), 1.96 – 1.85 (m, 2H), 1.69 (m, 12H), 1.54 – 1.45 (m, 6H), 1.40 – 1.32 (m, 3H), 1.31 – 1.25 (m, 3H), 1.01 (s, 3H), 0.74 (s, 3H). HRMS *m/z* 758.4063 [M + H]^+^.

**Ⅱ.** **Supplementary Figure legends**

**Fig. S1 Bufalin-induced growth inhibition of hepatoma cells.** HepG2, Huh7, SK-Hep-1 and Hepa3B cells were treated with the indicated concentrations of bufalin for 24 h. Cell proliferation was evaluated by MTT (**a-d**) and EdU (**e**) staining assays. Number of EdU-positive cells with green fluorescence was counted. Data are expressed as mean ± SD for three individual experiments at least. Scale bars＝200 μm. ^**^*P* < 0.01 *vs.* control group by ANOVA with Student’s *t*-test.

**Fig. S2 Bufalin** **causes the inhibition of hepatoma cell** **migration.** Huh7, SK-Hep-1 and Hepa3B cells were treated with the indicated concentrations of bufalin for 24 h and 48 h. Cell migration was detected by scratch wound healing (**a-c**) and transwell chamber assays (**d**). Cells stained with crystal violet were counted. Data are expressed as mean ± SD for three individual experiments at least. Scale bars＝200 μm. ^**^*P* < 0.01 *vs.* control group by ANOVA with Student’s *t*-test.

**Fig. S3 Binding of bufalin with SDC4 in** **Huh7 cells.** Streptavidin agarose affinity assay indicates that bufalin binds with SDC4 in Huh7 cells.

**Fig. S4 Bufalin promotes the interaction of SDC4 with DDX23.** Co-localization of DDX23 (red) and SDC4 (green) by immunofluorescence in HepG2 cell (Scale bars＝25 μm). The white arrows represent co-localization of SDC4 and DDX23 protein.

**Fig. S****5 Bufalin blocks MMPs, MAPK and EMT signaling pathways in HepG2 cells.** **a** Bufalin induces cell cycle arrest at G2/M phase in HepG2 cells. Cell cycle was investigated by flow cytometry, the proportion of bufalin treatment groups at G2/M phase was significantly increased compared to control groups. **b** Bufalin inhibits the enzymatic activity of MMP2 in HepG2 cells. Cells were treated with bufalin (10 nM) for 24 h. The enzymatic activity of MMP-2 was analyzed by gelatin zymography. **c**, **d** Bufalin blocks EMT signaling pathways in HepG2 cells. HepG2 cells were treated with DMSO or bufalin (10 nM) for 24 h, then fixed, stained with DAPI, anti-Cluadin-1 and anti-N-Cadherin antibodies, and observed by confocal microscopy (Scale bar = 10 μm).

**Fig. S6 IHC results of the expression of SDC4 and DDX23 in HCC and adjacent normal tissues.** The expression levels of SDC4/DDX23 were markedly increased in HCC tissues compared to adjacent normal tissues, as shown in representative immunohistochemical images and mean staining scores (Scale bar = 250 μm). The black arrows indicate positive staining cells.

**Fig. S7 Screening of siRNA** **oligo sequences for SDC4 in HepG2 cells.** Cells were transfected with the indicated siRNA oligo sequences or HA-SDC4 WT for 6 h, then cells were collected after 48 h for detecting the expression of SDC4 (**a**, **c**, **d**) and DDX23 (**b**).

**Fig. S8 siSDC4 or siDDX23 impairs EMT signaling pathways in HepG2 cells.** Snail, N-cadherin, TCF8/ZEB1, Vimentin and Claudin-1 were determined by western blot in HepG2 cells with SDC4 or DDX23 knockdown.

**Fig. S9 siSDC4 or siDDX23 antagonizes the inhibitory effect of** **bufalin on JNK/MAPK, cell cycle and MMPs signaling pathways in HepG2 cells.** CDK1, CyclinB1, P53, MMP2/9 and JNK were determined by western blot in HepG2 cells with SDC4 or DDX23 knockdown.

**Fig. S10 Synthetic scheme for biotin-bufalin.**

**Fig. S11 ^1^H NMR spectra for compound C3.**

**Fig. S12 ^1^H NMR spectra for compound C4.**

**Fig. S13 MS** **spectra for compound C4.**

**Fig. S14 ^1^H NMR spectra for compound C6.**

**Fig. S15 ^1^H NMR spectra for compound C7.**

**Fig. S16 MS spectra for compound C7.**

**Table S1. Target sequences used in this study.**

**Table S2. Correlations of expressions of SDC4 and DDX23 with clinicopathological features of HCC.**

**Table S3. Correlation of SDC4 and DDX23 expressed in HCC tissues (n = 41).**

**Ⅲ. Supplementary references**

^1^ Baschieri, A., Muzzioli, S., Fiorini, V., Matteucci, E., Massi, M. & Sambri, L., *et al*., Introducing a new family of biotinylated Ir(III)-Pyridyltriazole Lumophores: synthesis, photophysics, and preliminary study of avidin-binding properties. *ORGANOMETALLICS* **33** 6154 (2014).
