# Supplementary material for: Therapeutic potential of targeting membrane-spanning proteoglycan SDC4 in hepatocellular carcinoma
Source: Cell Death Dis. 2021 May 14;12(5):492. doi: 10.1038/s41419-021-03780-y (PMC8121893; doi:10.1038/s41419-021-03780-y)
Supplement: Supplementary file 2 — Supplementary Material [file 41419_2021_3780_MOESM2_ESM.docx]

**Table S1.** **Target sequences** **used in this study.**

| **Gene** | **Target Sequence** |
| --- | --- |
| SDC4-siRNA #1 | 5'-GCAGGAAUCUGAUGACUUUTT-3' |
| SDC4-siRNA #2 | 5'-GAGAAUCUCACCCGUUGAATT-3' |
| SDC4-siRNA #3 | 5'-UGCUCAUGUACCGUAUGAATT-3' |
| DDX23-siRNA #1 | 5'-CCUAAGGCCCAGCCAUUAUTT-3' |
| DDX23-siRNA #2 | 5'-GGAAACAGUUCCAAGACUUTT-3' |
| DDX23-siRNA #3 | 5'-GCACCAGGUGCAGUUGUUATT-3' |
