# Supplementary material for: Therapeutic potential of targeting membrane-spanning proteoglycan SDC4 in hepatocellular carcinoma
Source: Cell Death Dis. 2021 May 14;12(5):492. doi: 10.1038/s41419-021-03780-y (PMC8121893; doi:10.1038/s41419-021-03780-y)
Supplement: Supplementary file 3 — Supplementary Material [file 41419_2021_3780_MOESM3_ESM.docx]

**Table S2. Correlations of expressions of SDC4 and DDX23 with clinicopathological features of HCC.**

| Clinicopathological n SDC4 DDX23 |
| --- |
| features － ＋ Positive *P* － ＋ Positive *P* |
| rate rate |
| Age |
| ≤50 17 5 12 0.71 0.116 8 9 0.53 0.540 |
| ＞50 24 13 11 0.46 9 15 0.63 |
| Gender |
| Male 33 15 18 0.55 0.684 15 18 0.55 0.292 |
| Female 8 3 5 0.63 2 6 0.75 |
| Clinicopathological stages |
| I 11 9 2 0.36 0.012 8 3 0.36 0.009 |
| II 15 5 10 0.53 7 8 0.53 |
| III 15 4 11 0.73 2 13 0.80 |
| Hepatitis |
| Yes 9 4 5 0.56 0.970 5 4 0.44 0.331 |
| No 32 14 18 0.56 12 20 0.63 |

Data were analyzed by the chi-square; + positive expression, − negative expression.
