# Supplementary material for: Therapeutic potential of targeting membrane-spanning proteoglycan SDC4 in hepatocellular carcinoma
Source: Cell Death Dis. 2021 May 14;12(5):492. doi: 10.1038/s41419-021-03780-y (PMC8121893; doi:10.1038/s41419-021-03780-y)
Supplement: Supplementary file 4 — Supplementary Material [file 41419_2021_3780_MOESM4_ESM.docx]

**Table S3. Correlation of SDC4 and DDX23 expressed in HCC tissues (n = 41).**

| SDC4 *R P* |
| --- |
| － ＋  DDX23 |
| － 13 4 0.598 ＜0.001 |
| ＋ 4 20 |

Data were analyzed by the Pearson correlation analysis; + positive expression, − negative expression.
