# Supplementary figures and images for: Therapeutic potential of targeting membrane-spanning proteoglycan SDC4 in hepatocellular carcinoma
Source: Cell Death Dis. 2021 May 14;12(5):492. doi: 10.1038/s41419-021-03780-y (PMC8121893; doi:10.1038/s41419-021-03780-y)

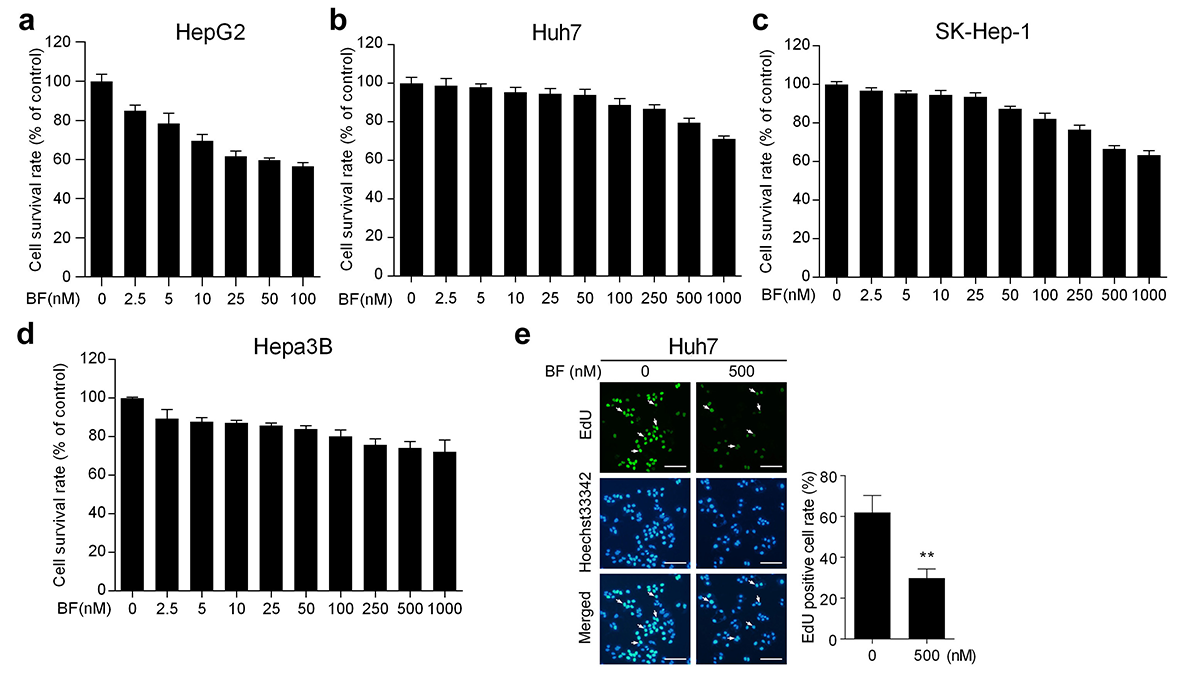

Supplement: Supplementary file 5 — Supplementary Material [file 41419_2021_3780_MOESM5_ESM.tif]

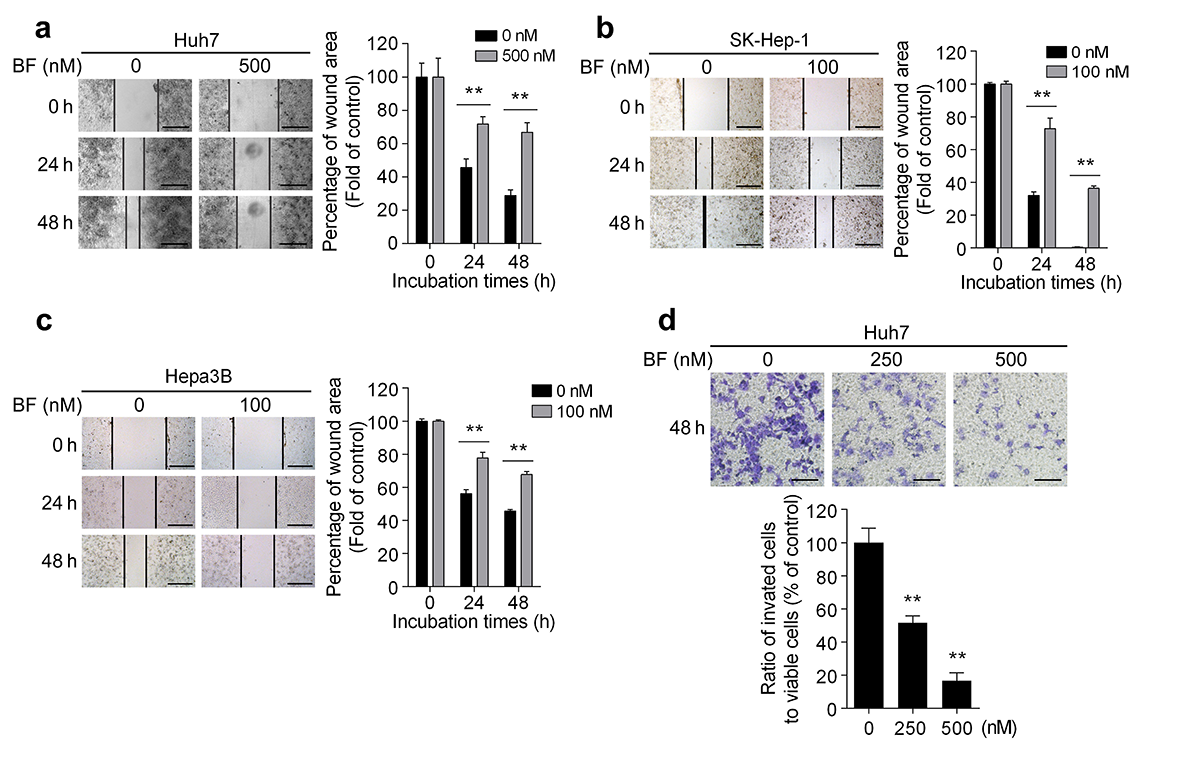

Supplement: Supplementary file 6 — Supplementary Material [file 41419_2021_3780_MOESM6_ESM.tif]

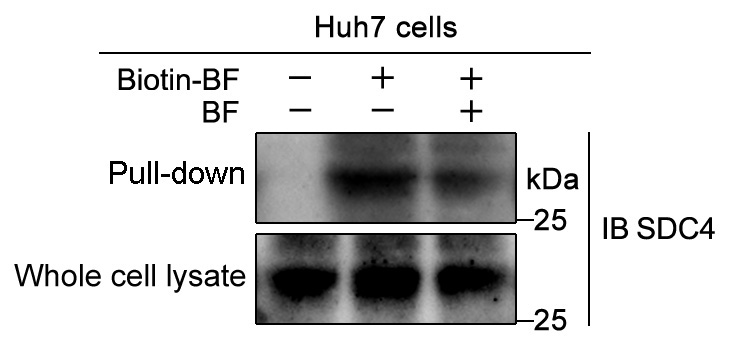

Supplement: Supplementary file 7 — Supplementary Material [file 41419_2021_3780_MOESM7_ESM.tif]

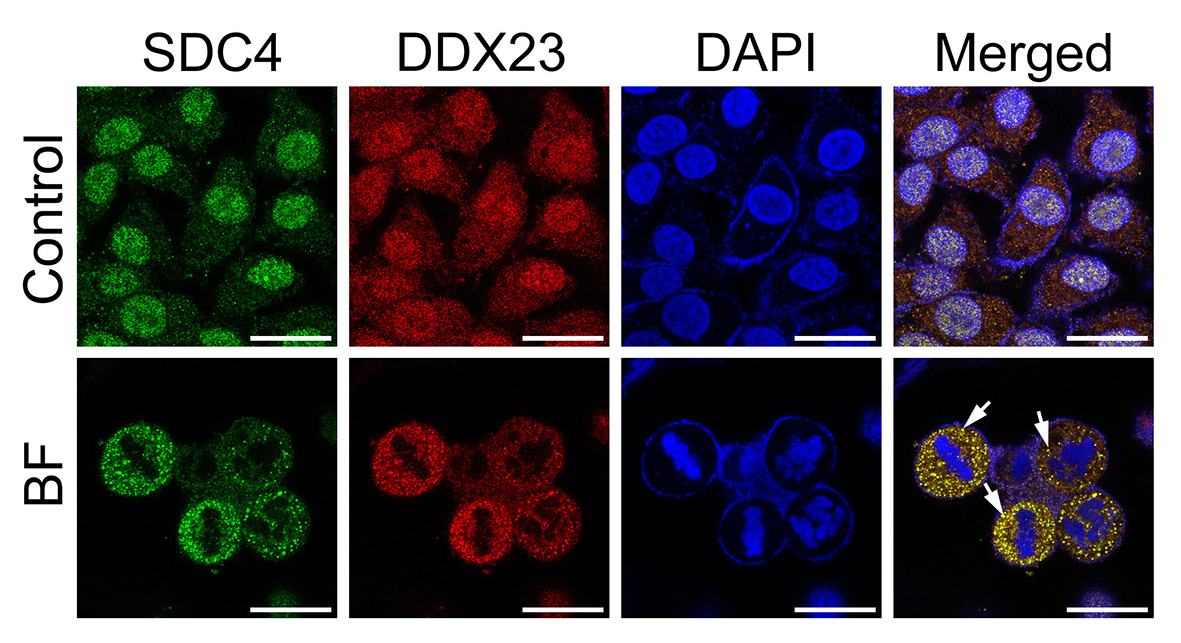

Supplement: Supplementary file 8 — Supplementary Material [file 41419_2021_3780_MOESM8_ESM.tif]

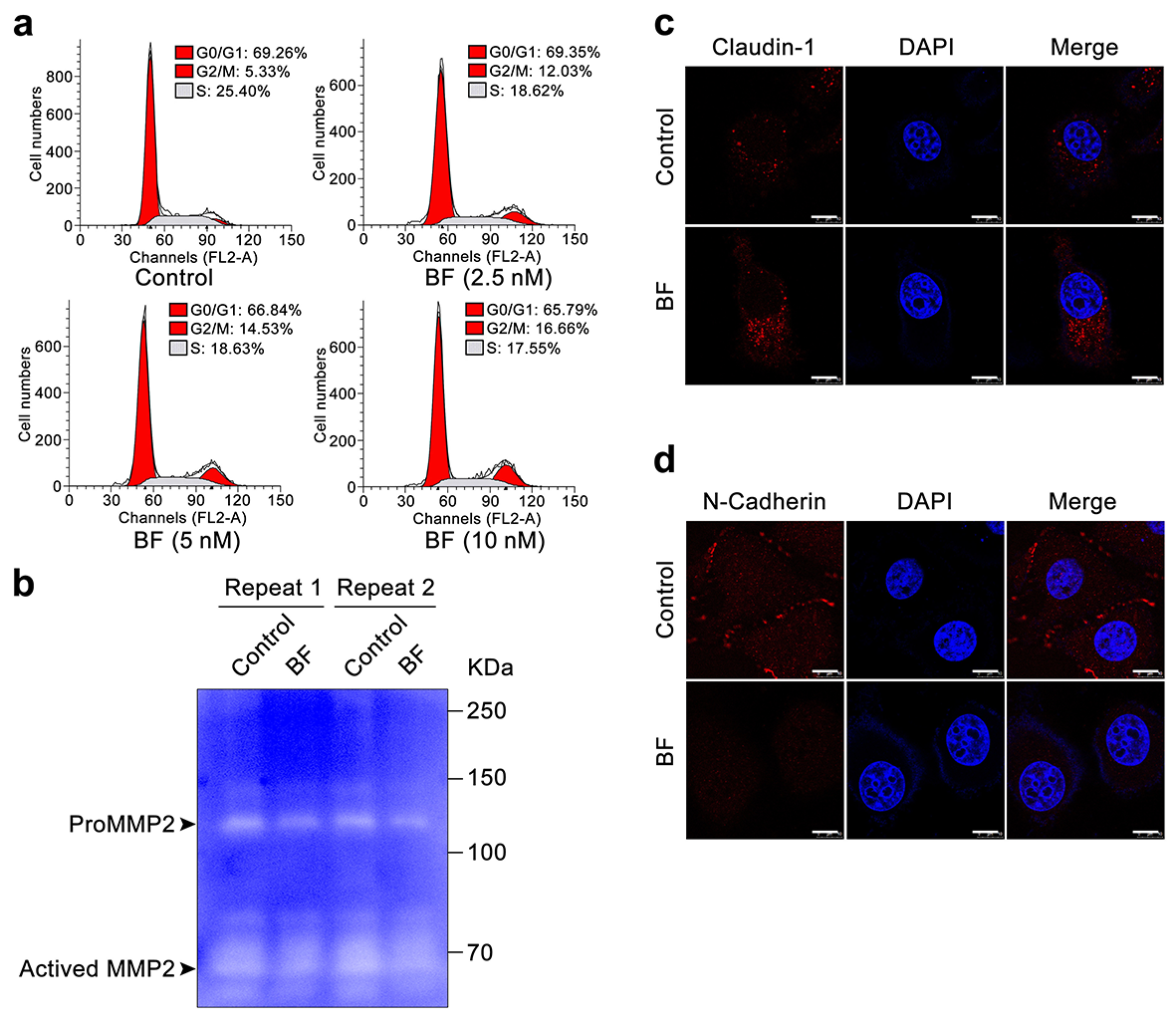

Supplement: Supplementary file 9 — Supplementary Material [file 41419_2021_3780_MOESM9_ESM.tif]

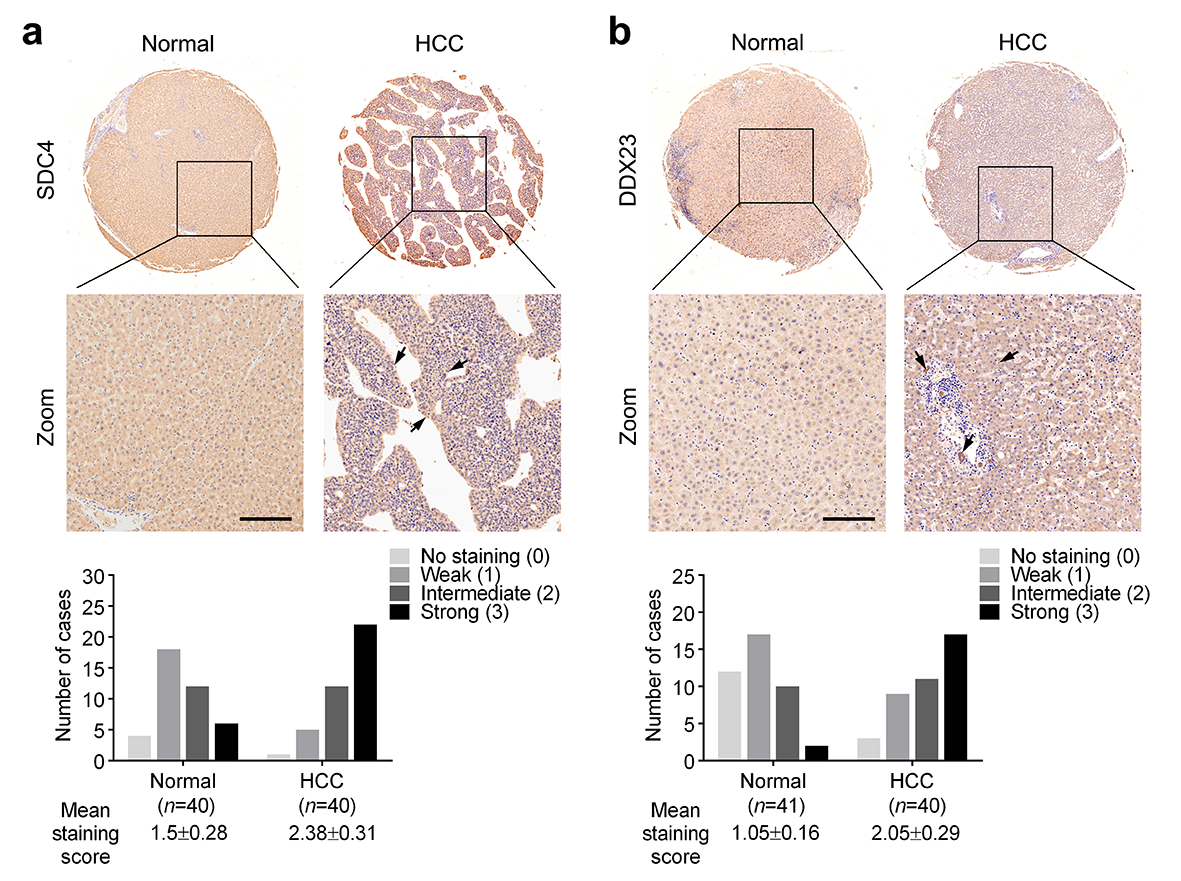

Supplement: Supplementary file 10 — Supplementary Material [file 41419_2021_3780_MOESM10_ESM.tif]

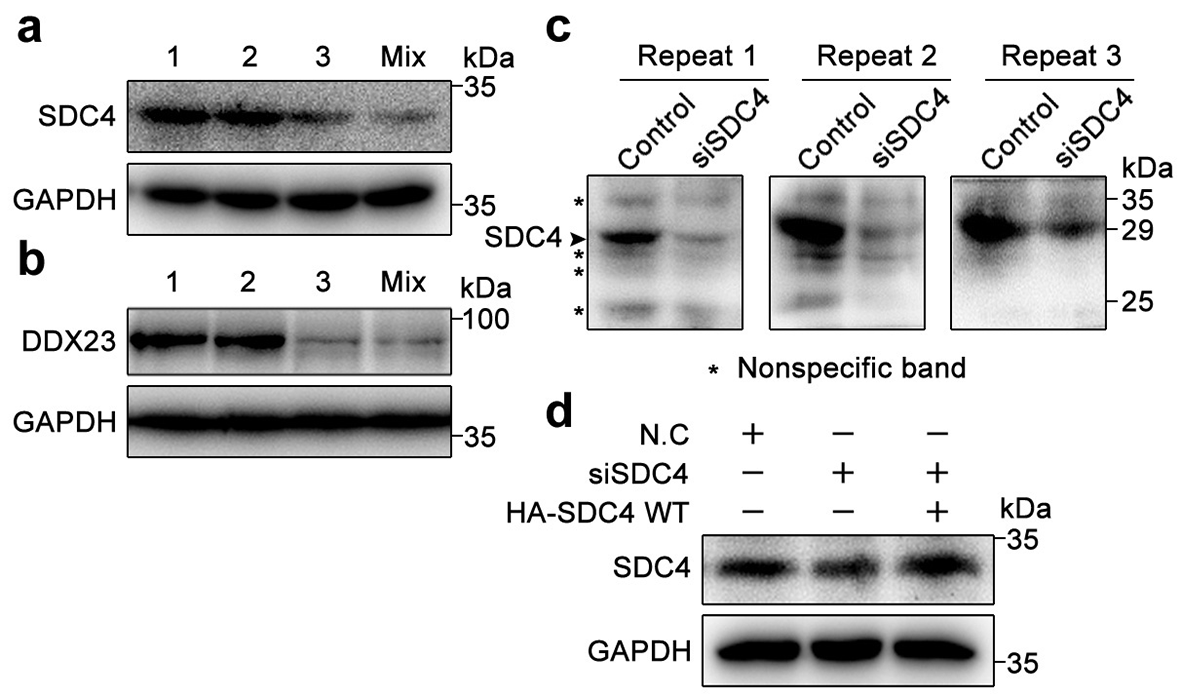

Supplement: Supplementary file 11 — Supplementary Material [file 41419_2021_3780_MOESM11_ESM.tif]

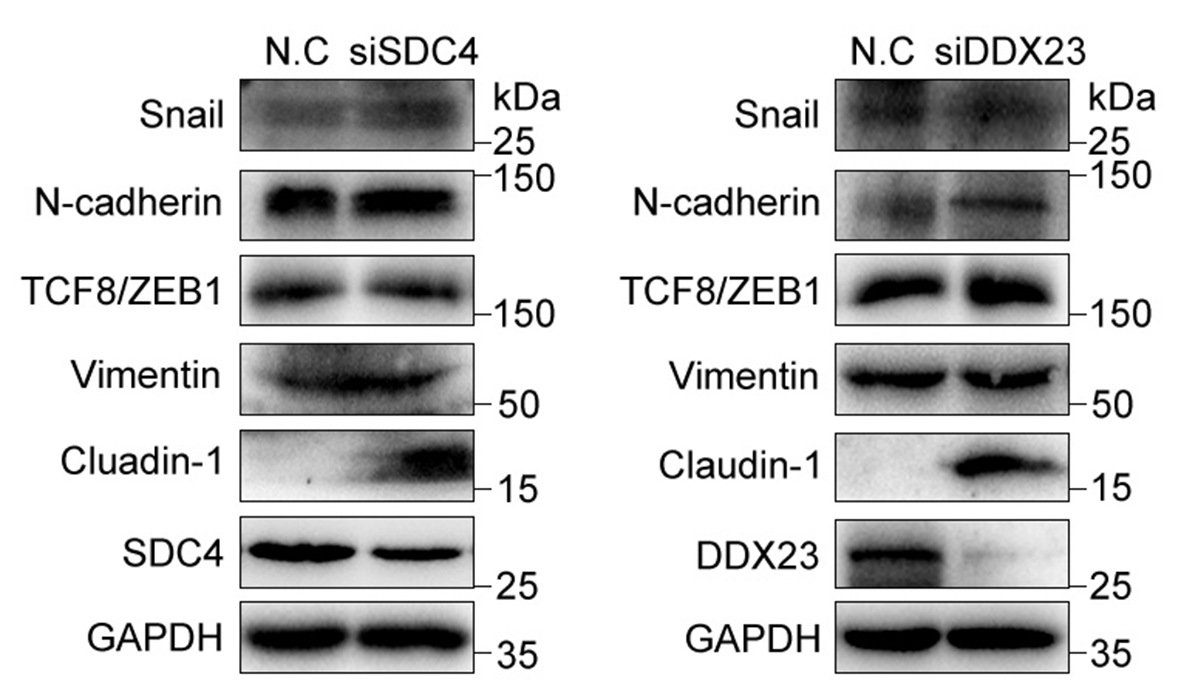

Supplement: Supplementary file 12 — Supplementary Material [file 41419_2021_3780_MOESM12_ESM.tif]

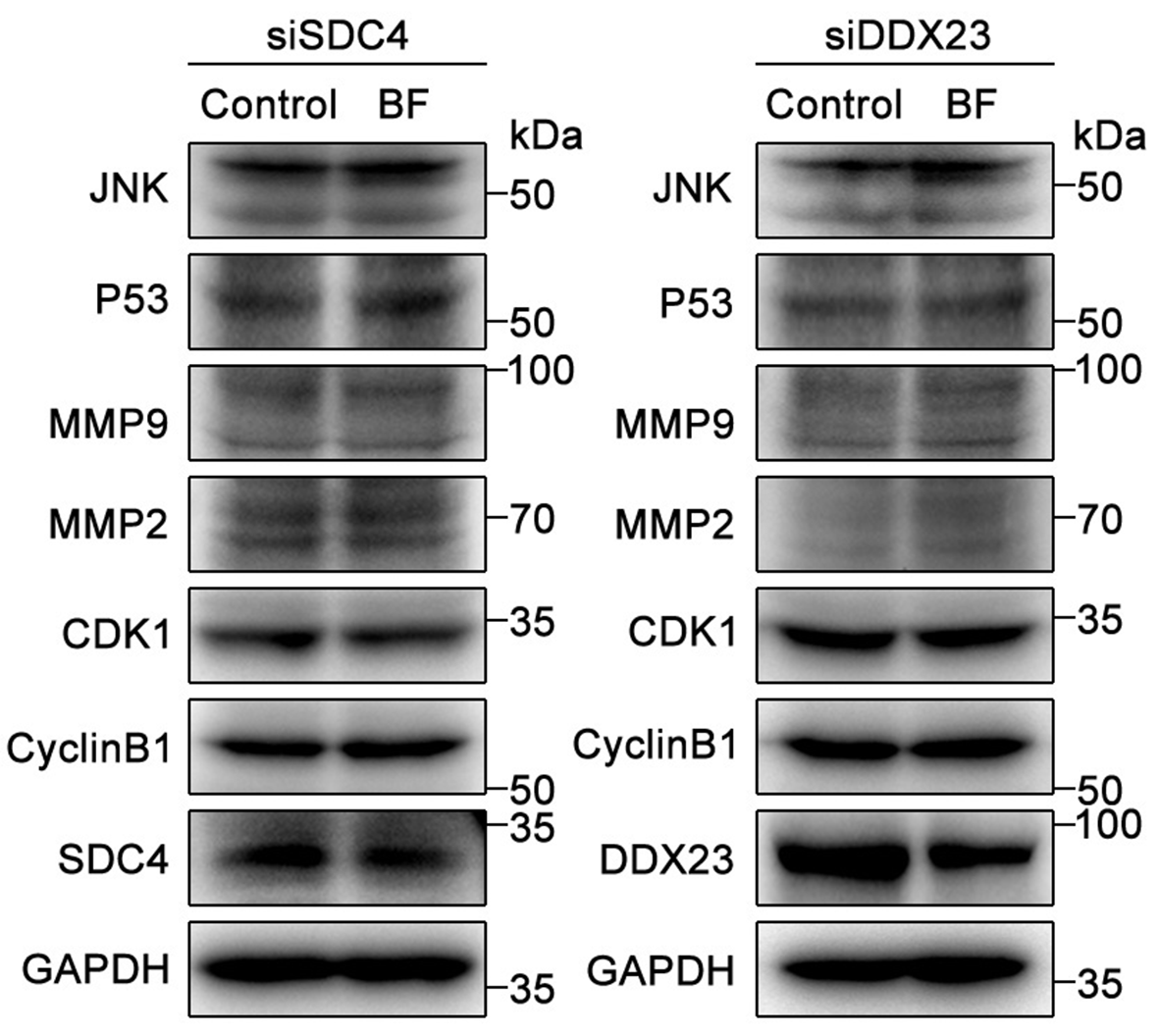

Supplement: Supplementary file 13 — Supplementary Material [file 41419_2021_3780_MOESM13_ESM.tif]

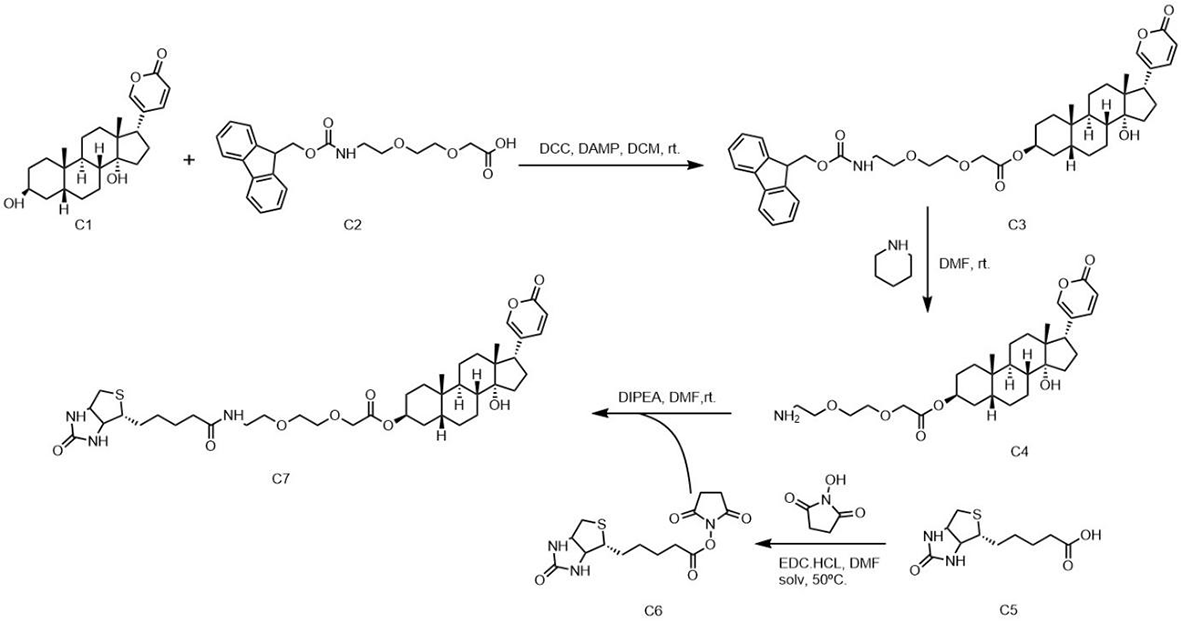

Supplement: Supplementary file 14 — Supplementary Material [file 41419_2021_3780_MOESM14_ESM.tif]

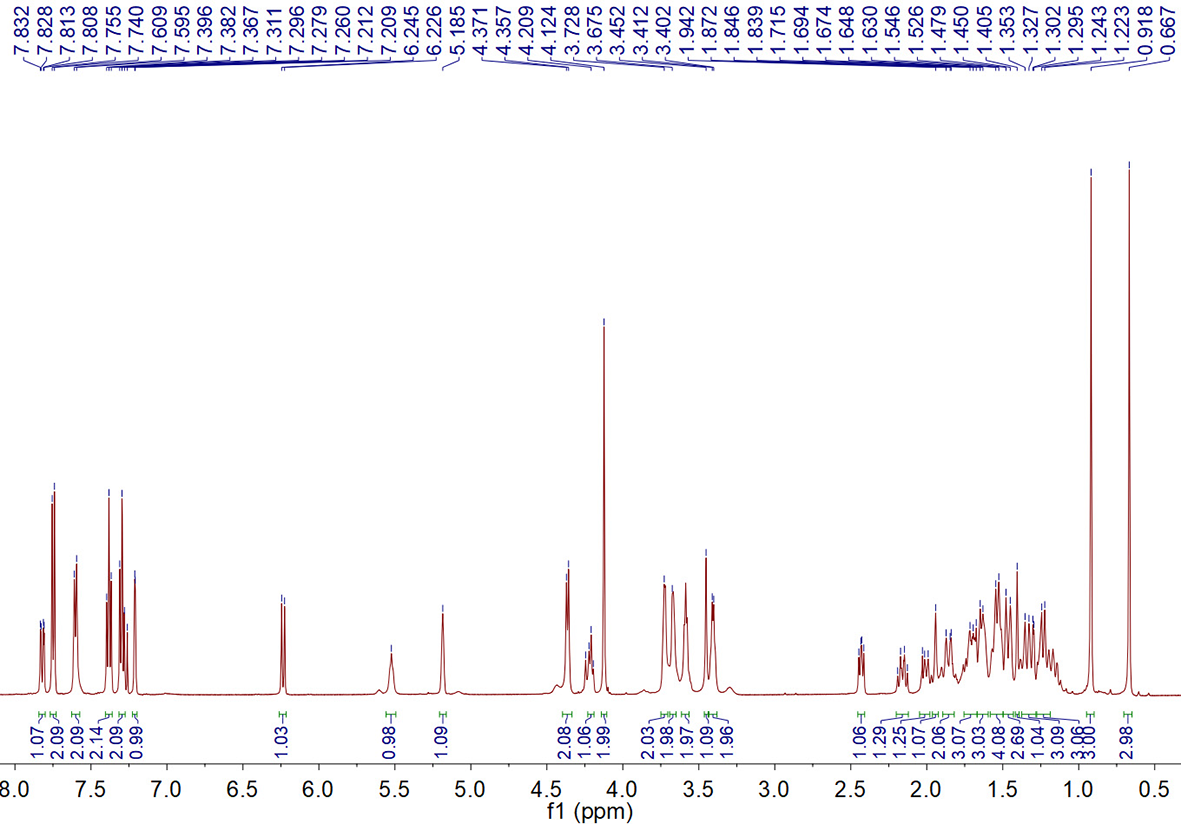

Supplement: Supplementary file 15 — Supplementary Material [file 41419_2021_3780_MOESM15_ESM.tif]

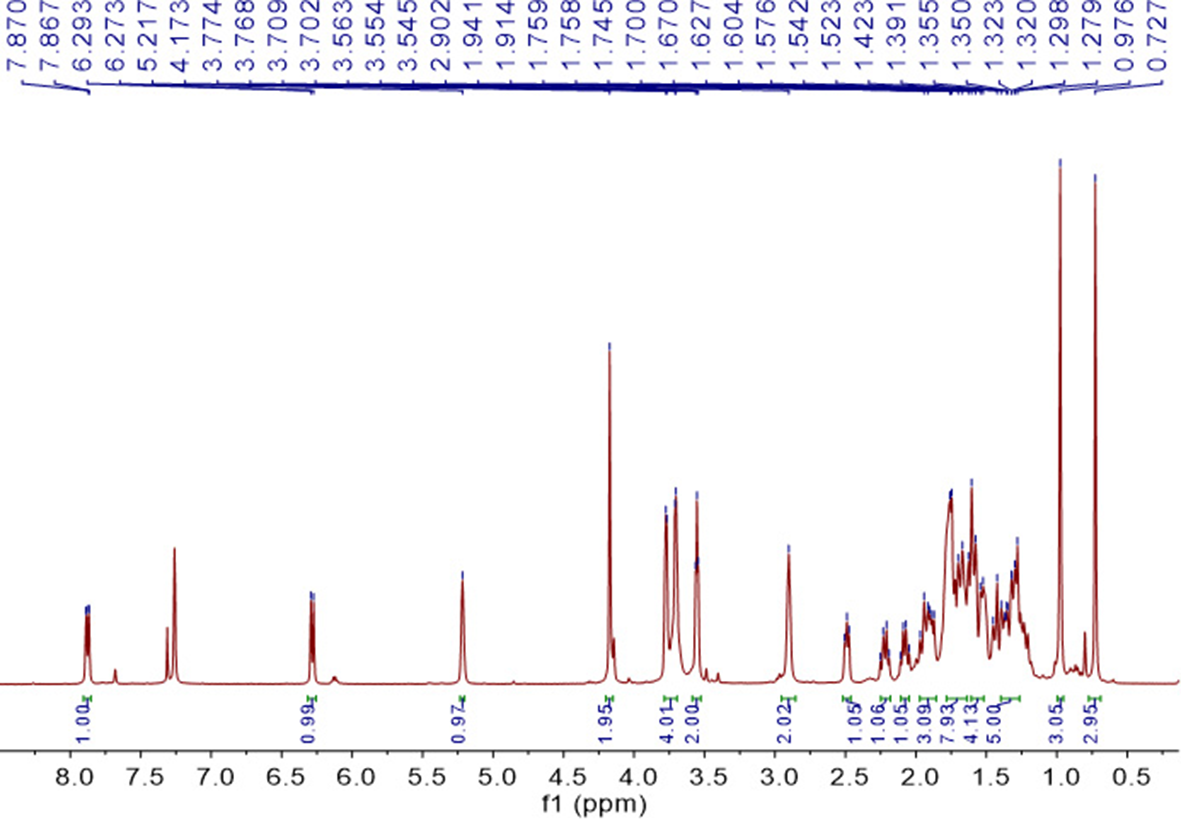

Supplement: Supplementary file 16 — Supplementary Material [file 41419_2021_3780_MOESM16_ESM.tif]

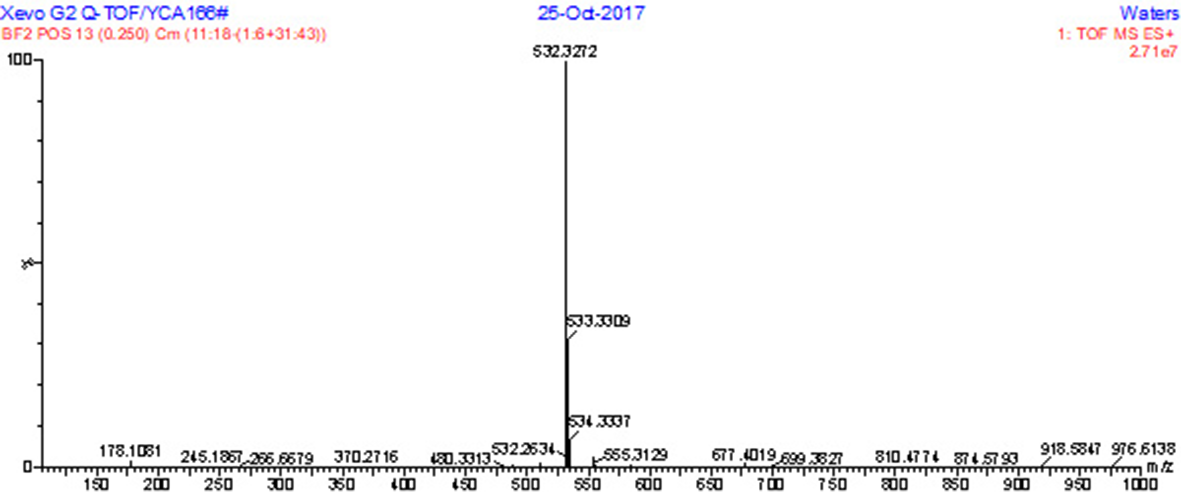

Supplement: Supplementary file 17 — Supplementary Material [file 41419_2021_3780_MOESM17_ESM.tif]

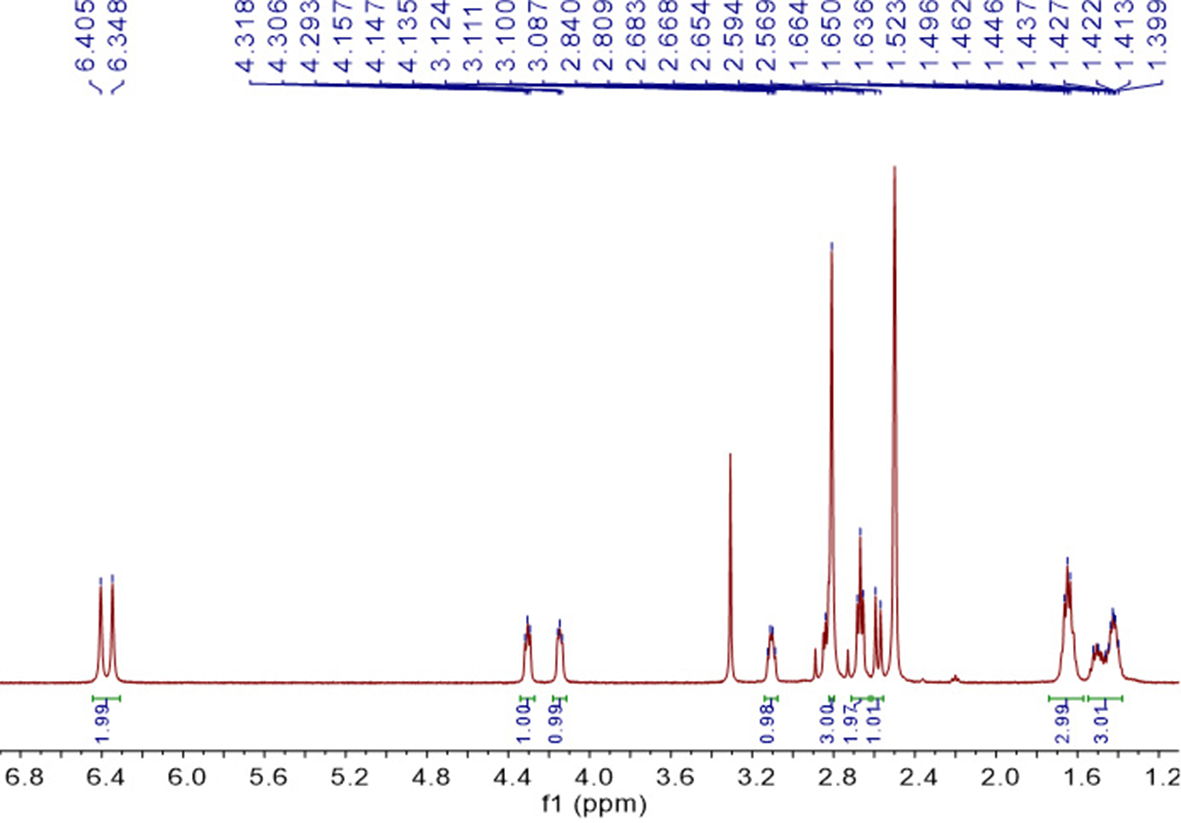

Supplement: Supplementary file 18 — Supplementary Material [file 41419_2021_3780_MOESM18_ESM.tif]

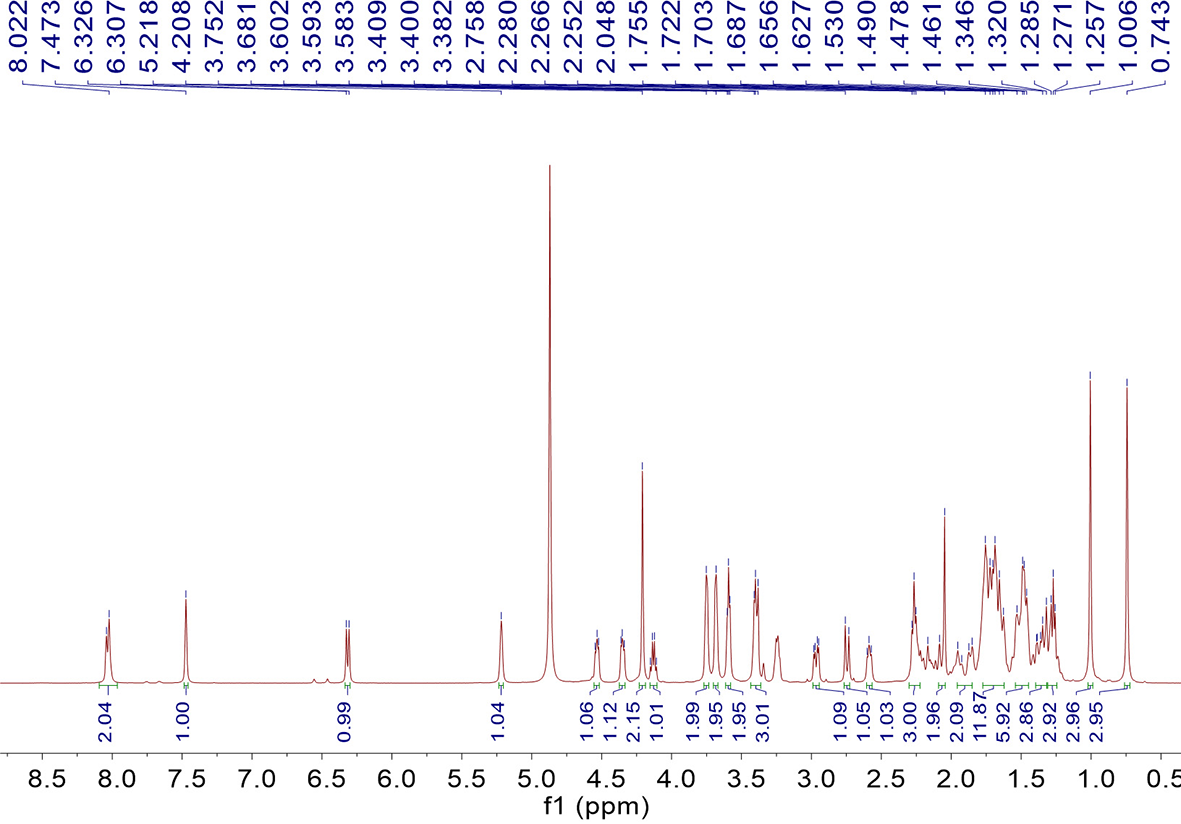

Supplement: Supplementary file 19 — Supplementary Material [file 41419_2021_3780_MOESM19_ESM.tif]

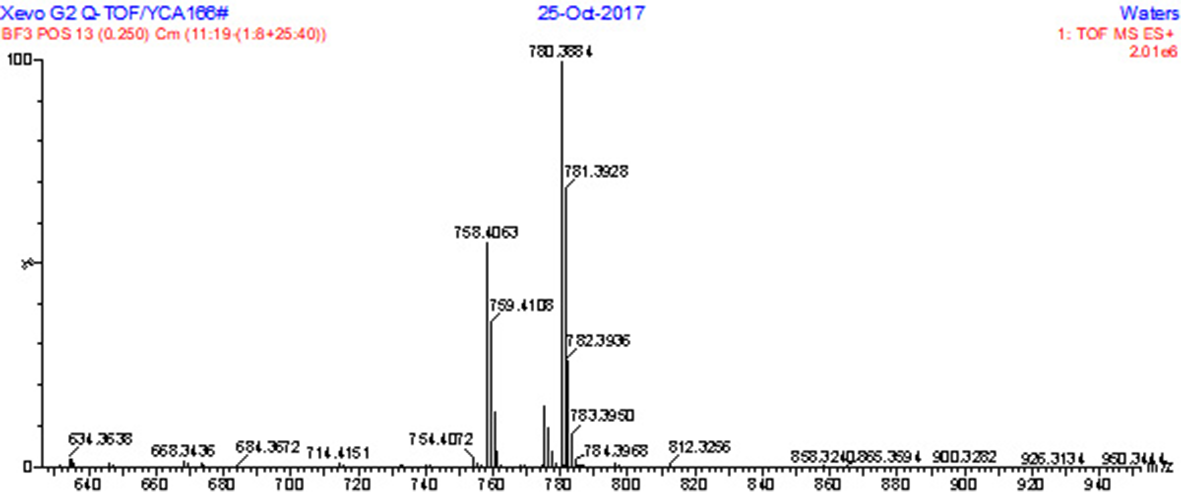

Supplement: Supplementary file 20 — Supplementary Material [file 41419_2021_3780_MOESM20_ESM.tif]
